# Instrumental variable estimation for compositional treatments

**DOI:** 10.1038/s41598-025-89204-9

**Published:** 2025-02-12

**Authors:** Elisabeth Ailer, Christian L. Müller, Niki Kilbertus

**Affiliations:** 1 Helmholtz Munich, Ingolstädter Landstraße 1, 85764 Neuherberg, Germany; 2 TUM School of Computation, Information and Technology, Technical University of Munich, Boltzmannstraße 3, 85748 Garching, Germany; 3 Munich Center for Machine Learning (MCML), Munich, Germany; 4 Department of Statistics, Ludwig-Maximilian University, Geschwister-Scholl-Platz 1, 80539 Munich, Germany; 5 Center for Computational Mathematics, Flatiron Institute, 162 5th Ave, 10010 New York, NY United States

**Keywords:** Causality, Cause-effect estimation, Compositional data, Instrumental variable, Microbial diversity, Computational biology and bioinformatics, Mathematics and computing

## Abstract

Many scientific datasets are compositional in nature. Important biological examples include species abundances in ecology, cell-type compositions derived from single-cell sequencing data, and amplicon abundance data in microbiome research. Here, we provide a causal view on compositional data in an instrumental variable setting where the composition acts as the cause. First, we crisply articulate potential pitfalls for practitioners regarding the interpretation of compositional causes from the viewpoint of interventions and warn against attributing causal meaning to common summary statistics such as diversity indices in microbiome data analysis. We then advocate for and develop multivariate methods using statistical data transformations and regression techniques that take the special structure of the compositional sample space into account while still yielding scientifically interpretable results. In a comparative analysis on synthetic and real microbiome data we show the advantages and limitations of our proposal. We posit that our analysis provides a useful framework and guidance for valid and informative cause-effect estimation in the context of compositional data.

## Introduction and motivation

The statistical modeling of compositional (or relative abundance) data plays a pivotal role in many areas of science, ranging from the analysis of mineral samples or rock compositions in earth sciences^1^ to correlated topic modeling in large text corpora^2,3^. Recent advances in biological high-throughput sequencing techniques, including single-cell RNA-Seq and microbial amplicon sequencing^4,5^, have triggered renewed interest in compositional data analysis. Since only a limited total number of transcripts can be captured in a sample by current sequencing technologies, the resulting count data provides relative abundance information about mRNA transcripts or microbial amplicon sequences, respectively^6,7^.

For example, in microbiome sequencing, this stems from the fact that one cannot easily control for the total number of microbes entering the measurement process. Bacterial microbiome measurements typically come in the form of counts of operational taxonomic units (OTUs) or amplicon sequencing variants (ASVs) derived from high-throughput sequencing of 16S ribosomal RNA (rRNA)^8^ and are summarized as taxonomic compositions, e.g., on the species, genus, or family level.

One way of dealing with the available relative abundance information is to normalize read counts by their respective totals, resulting in *compositional data*. Compositional data comprises the proportions of some whole, implying that data points live on the unit simplex $\mathbb {S}^{p-1} := \{x \in \mathbb {R}^p_{\ge 0} \mid \sum _{j=1}^p x_j = 1\}$.

In the microbiome example, assume there are *p* different microbial taxa that have been identified in a human gut microbiome experiment. A specific gut microbiome measurement is then represented by a vector *x*, where $x_j$ denotes the relative abundance of taxon *j* (under an arbitrary ordering of taxa). An increase in $x_1$ within this composition could correspond to an actual increase in the absolute abundance of the first taxon, while the rest remained constant. However, it could equally result from a decrease of the absolute abundance of the first species with the remaining ones having decreased even more.

Statisticians have recognized the significance of compositional data early on (dating back to Karl Pearson) and tailored models to naturally account for compositionality via simplex arithmetic^1^. Despite these efforts, adjusting predictive statistical and machine learning methods to compositional data remains an active field of research^9–18^.

This work focuses on estimating the causal effect of a composition on a categorical or continuous outcome. Only recently have the fundamental challenges in interpreting causal effects of compositions been acknowledged explicitly^19,20^ with little work on how to estimate such effects from observational data. Our work provides scalable methods that *enable practitioners to answer the simple question: “What is the causal effect of a composition on some outcome of interest?”* In the following, we develop methods which can solve this causal questions while also creating an understanding of common pitfalls in causal settings.

### Pitfalls with summary statistics

First, let us motivate the *compositional* aspect of the question. In microbiome research specifically, species diversity became the center of attention to an extent that asking “what is the causal effect of *the diversity* of a composition *X* on the outcome *Y*?” appears more intuitive than asking for the causal effect of individual abundances. In fact, popular books and research articles alike seem to suggest that (bio-)diversity is indeed an important *causal driver* of ecosystem functioning and human health, even though these claims are largely grounded in observational, non-experimental data^21,22^. Similar summary statistics or low-dimensional representations have been proposed in other domains such as in single-cell RNA data^23^. We now explain why, even in situations where summary statistics appear to be useful proxies, no causal conclusions can be drawn from them.

Let us consider $\alpha$-diversity as an example of a one-dimensional summary statistic of a microbiome measurement, e.g. $\alpha _{\text {Simpson}} = -\sum _{j = 1}^p (x_j)^2$ or $\alpha _{\text {Shannon}} = -\sum _{j=1}^p x_j \log x_j$. The “causal effect” of the diversity $\alpha$ on some outcome of interest *Y* (e.g., health or disease indicator) is usually considered to be the expected value of *Y* under an *intervention on the diversity*, i.e., externally setting the diversity to a chosen value $\alpha ^*$, with all host and environmental factors unchanged. This causal effect is commonly denoted by ${{\,\mathrm{\mathbb {E}}\,}}[Y \mid do(\alpha =\alpha ^*)]$. When ${{\,\mathrm{\mathbb {E}}\,}}[Y \mid do(\alpha = \alpha _1)] < {{\,\mathrm{\mathbb {E}}\,}}[Y \mid do(\alpha = \alpha _2)]$ for two diversity values $\alpha _1, \alpha _2$ with $\alpha _1 < \alpha _2$, one would then be tempted to conclude that “increasing diversity $\alpha$ causes an increase in the outcome *Y*”, which is often loosely translated to “diversity is a causal driver for health”. We now highlight critical issues with this approach.

**Fig. 1:**
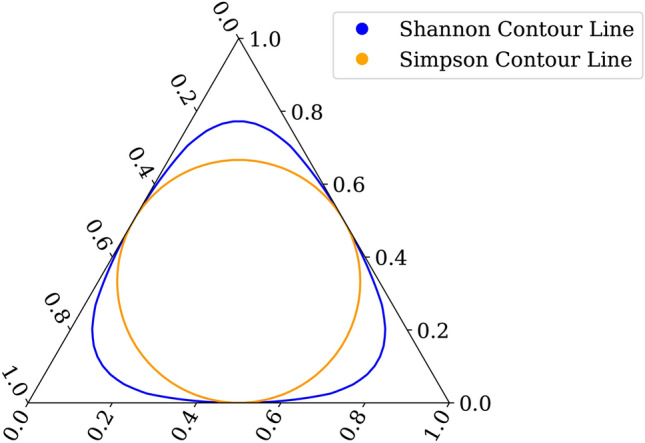
The ternary plot shows an exemplary scenario with $p=3$. The orange contour contains compositions for which the Simpson diversity is constant, while the blue contour shows compositions for which the Shannon diversity is constant. Shannon diversity changes along contours of Simpson diversity and vice versa.

(a) When considering the proposed causal effect estimand ${{\,\mathrm{\mathbb {E}}\,}}[Y \mid do(\alpha =\alpha ^*)]$ directly, one presupposes the existence of clearly defined interventions on $\alpha$. However, there are infinitely many ways of changing the diversity of a composition by a fixed amount. This ‘many-to-one’ nature prevents a consistent conceptualization of external interventions. In particular, for a given value of $\alpha$, there is a $(p-2)$-dimensional subspace of $\mathbb {S}^{p-1}$ with that value of $\alpha$. Hence, an intervention to “increase the diversity of a given composition” by some $\Delta \alpha$ is highly ambiguous. The different ways of achieving this change must be expected to have different implications for the outcome *Y*. Similarly, most common diversity measures are invariant under permutations of components and the above approach would require us to conclude that all *p*! permutations of a composition are functionally completely equivalent with regard to the outcome *Y*—an abstruse claim. *Hence, assigning causal powers to diversity by estimating* ${{\,\mathrm{\mathbb {E}}\,}}[Y \mid do(\alpha )]$ *is highly ambiguous and does not carry the intended meaning.* This concern is further exacerbated by the difficulty and ambiguity in measuring $\alpha$-diversity in the first place^7,24,25^.

(b) The definition of $\alpha$-diversity is not unique, which could lead to a potential search for positive results by using a different metric^26^ or contradictory causal claims. Consider two different one-dimensional summary statistics $\alpha _1, \alpha _2$ on $\mathbb {S}^{p-1}$. These can be defined in terms of their contours, i.e., the collection of $(p-2)$-dimensional subspaces of $\mathbb {S}^{p-1}$ of constant values of $\alpha _1$ and $\alpha _2$ respectively. Since they are different, there will be a contour line of $\alpha _1$ along which $\alpha _2$ either increases or decreases. Along this path through compositions, we would have to conclude that the causal effect of one summary statistic is zero, while it is non-zero for the other. See Fig. 1 for a visualization. In typical scenarios, there is no “one correct” summary statistic, such that reliable claims even about the sign of the causal effect of a summary statistic of a composition become void.

### Cause-effect estimation with instrumental variables

While researchers continue to develop predictive methods for compositional data^16^, in most scientific contexts causal effects are of greater interest. For example, the human microbiome co-evolves with its host and the external environment through diet, activity, climate, or geography, etc. leading to plentiful microbiome-host-environment interactions^27^. Carefully designed studies may allow us to control for certain environmental factors and specifics of the host. In fact, several recent works studied the causal mediation effect of the microbiome on health-related outcomes, assuming all relevant covariates are observed and can be controlled for^28–34^, or vice versa, the effect of environmental factors on the microbiome^35^. However, in practice there is little hope of measuring *all* latent factors in these complex interactions. In such a situation, a purely predictive model will suffer from bias due to the unobserved confounders. Such unobserved confounders are a major hurdle in cause-effect estimation broadly and also specifically for compositional causes.

Concretely, without further assumptions, the direct causal effect $X \rightarrow Y$ is not identified from observational data in the presence of unobserved confounding $X \leftarrow U \rightarrow Y$^36^. One common way to still identify the causal effect from purely observational data is through so-called instrumental variables (IV)^37^. An *instrumental variable* *Z* is a variable that has an effect on the cause *X* ($Z \rightarrow X$), but is independent of the confounder ($Z {{\,\mathrm{\perp \!\!\!\perp }\,}}U$), and conditionally independent of the outcome given the cause and the confounder ($Z {{\,\mathrm{\perp \!\!\!\perp }\,}}Y \mid \{U, X\}$). In practice, it can be hard to find valid instruments for a target effect^38^, but when they do exist, instrumental variables often render efficient cause-effect estimation possible.

In this work, we develop interpretable methods to estimate the direct causal effect of a *compositional cause* *X* on a continuous or categorical outcome *Y* within the IV setting. The question of whether and how cause-effect estimation for compositional treatments under unobserved confounding is possible remains unanswered in the literature, motivating our in-depth analysis of two-stage methods for interpretable cause-effect estimation of individual relative abundances on the outcome. In the analysis, we focus on a careful selection and combination of existing approaches and a thorough examination of potential pitfalls and mis-usage. Our extensive empirical evaluations carefully assess assumptions (additive noise, strong instruments) and model misspecification as a potential obstacle to interpretable and reliable effect estimates. We evaluate the efficacy and robustness of our proposed methods on both synthetic and real data from a mouse experiment, examining how the gut microbiome (*X*) affects body weight (*Y*) instrumented by sub-therapeutic antibiotic treatment (STAT) (*Z*).

The rest of the manuscript proceeds as follows. First, we introduce the concepts of compositional data and instrumental variables in detail. Following this introduction of our methods, we provide some simulation to study the advantage and potential pitfalls in using high-dimensional compositional data in instrumental variable settings. Last but not least, we then apply the methods to a real world dataset.

## Methods

**Fig. 2:**
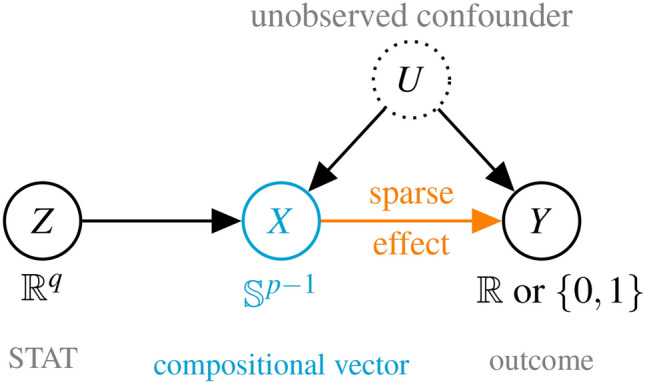
Cause-effect estimation of $X \rightarrow Y$ via an instrumental variable *Z* for compositional *X*.

### Instrumental variables

We briefly recap the assumptions of the instrumental variable setting as depicted in Fig. 2. For an *outcome* (or *effect*) *Y*, a *treatment* (or *cause*) *X*, and potential *unobserved confounders* *U*, we assume access to a discrete or continuous *instrument* $Z \in \mathbb {R}^q$ satisfying (i) $Z {{\,\mathrm{\perp \!\!\!\perp }\,}}U$ (the confounder is independent of the instrument), (ii) $Z {{\,\mathrm{\not \perp \!\!\!\perp }\,}}X$ (“the instrument influences the cause”), and (iii) $Z {{\,\mathrm{\perp \!\!\!\perp }\,}}Y \mid \{X, U\}$ (“the instrument influences the outcome only through the cause”). Our goal is to estimate the direct causal effect of *X* on *Y*, written as ${{\,\mathrm{\mathbb {E}}\,}}[Y | do(x)]$ in the do-calculus notation^36^ or as ${{\,\mathrm{\mathbb {E}}\,}}[Y(x)]$ in the potential outcome framework^39^, where *Y*(*x*) denotes the potential outcome for treatment value *x*. The functional dependencies are $X = g(Z,U)$, $Y = f(X,U)$. While *Z*, *X*, *Y* denote random variables, we also consider a dataset of *n* i.i.d. samples $\mathscr {D}= \{(z_i, x_i, y_i)\}_{i=1}^n$ from their joint distribution. We arrange observations in matrices or vectors denoted by $\varvec{X}\in \mathbb {R}^{n\times p}$ or $\varvec{X}\in (\mathbb {S}^{p-1})^n$, $\varvec{Z}\in \mathbb {R}^{n \times q}$, $\varvec{y}\in \mathbb {R}^n$.

Without further restrictions on *f* and *g*, the causal effect is not identified^40–42^. The most common assumption leading to identification is that of *additive noise*, namely $Y = f(X) + U$ with ${{\,\mathrm{\mathbb {E}}\,}}[U] = 0$ but not necessarily $X {{\,\mathrm{\perp \!\!\!\perp }\,}}U$. Here, we overload the symbols *f* and *g* for simplicity. The implied Fredholm integral equation of first kind ${{\,\mathrm{\mathbb {E}}\,}}[Y \mid Z] = \int f(x)\, \textrm{d}P(X\mid Z)$ is generally ill-posed. While the linear case is well understood^37^, under certain regularity conditions the IV problem can be solved consistently even for non-linear *f*, see e.g.,^43,44^ and more recently^45–48^. However, in this case the problem is typically under-specified in that multiple *f* are compatible with the observed data and regularization techniques are typically used to obtain a unique solution—typically the smallest compatible *f* according to some norm. It is thus difficult to interpret estimates of non-linear causal-effects in a way that aids understanding of the underlying processes.

In the simplest case, where $X \in \mathbb {R}^p$ and *f*, *g* are linear, a standard *instrumental variable estimator* is

1 $$\begin{aligned} \hat{\beta }_{\textrm{iv}}= (\varvec{X}^T \varvec{P}_{Z} \varvec{X})^{-1} \varvec{X}^T \varvec{P}_{Z}\, \varvec{y}\end{aligned}$$

with $\varvec{P}_{Z} = \varvec{Z}(\varvec{Z}^T \varvec{Z})^{-1} \varvec{Z}^T$^37^. For the *just-identified* case $q=p$ as well as the over-identified case $q > p$, this estimator is consistent and asymptotically unbiased, albeit not unbiased. In the *under-identified* case $q < p$, where there are fewer instruments than treatments, the orthogonality of *Z* and *U* does not imply a unique solution. Again, regularization or other objectives such as sparsity assumptions have been proposed to obtain unique a unique solution within the space of compatible $\beta$^49–51^. The estimator $\hat{\beta }_{\textrm{iv}}$ can also be interpreted as the outcome of a *two-stage least squares* (2SLS) procedure consisting of (1) regressing $\varvec{X}$ on $\varvec{Z}$ via OLS $\hat{\delta } = (\varvec{Z}^T \varvec{Z})^{-1} \varvec{Z}^T \varvec{X}$, and (2) regressing $\varvec{y}$ on the predicted values $\hat{\varvec{X}} = \varvec{Z}\hat{\delta }$ via OLS, again resulting in $\hat{\beta }_{\textrm{iv}}$. Practitioners are typically discouraged from using the manual two-stage approach, because the OLS standard errors of the second stage are wrong—a correction is needed^37^. However, we note that the point estimator obtained by the manual two-stage procedure is equivalent to Eq. (1).

Moreover, the two-stage description suggests that the two-stages are independent problems and thereby seems to invite us to mix and match different regression methods as we see fit.

The authors in^37^ highlight that the asymptotic properties of $\hat{\beta }_{\textrm{iv}}$ rely on the fact that for OLS the residuals of the first stage are uncorrelated with the instruments $\varvec{Z}$. Hence, for OLS we achieve consistency of $\hat{\beta }_{\textrm{iv}}$ *even when the first stage is misspecified*. For a non-linear first stage regression we may only hope to achieve uncorrelated residuals asymptotically when the model is correctly specified. Replacing the OLS first stage with a non-linear model is known as the “forbidden regression”, a term commonly attributed to Prof. Jerry Hausmann.

Angrist and Pischke acknowledge that the practical relevance of the forbidden regression is not well understood. However, the rather strong term tries to warn against a careless use of two consecutive regression models for the two stages. First, when also the second stage is assumed to be non-linear, one would require independence of the first stage residuals from *Z*. Secondly, if this requirement could not be guaranteed, both models would have to be specified correctly. Something that—in practice—can seldom be guaranteed. Nevertheless, nonlinear models for the IV setting are definitely feasible.

Starting with^52^ there is now a rich literature on the circumstances under which “manual 2SLS” with non-linear first (and/or second) stage can yield consistent causal estimators. Primarily interested in *compositional* treatments *X*, we cannot directly use OLS for either stage. Since there is no theoretical guidance for this case, we assess our options empirically, paying great attention to potential issues due to the “forbidden regression” and misspecification in our proposed methods.

### Compositional data

#### Simplex geometry

The authors in^1^ introduced the *perturbation* and *power transformation* as the simplex $\mathbb {S}^{p-1}$ counterparts to addition and scalar multiplication of Euclidean vectors in $\mathbb {R}^p$:

$$\begin{aligned} \begin{array}{l|c} \begin{array}{c} \hbox {Perturbation} \\ \oplus : \mathbb {S}^{p-1} \times \mathbb {S}^{p-1} \rightarrow \mathbb {S}^{p-1} \\ x \oplus w = C(x_1 w_1, \ldots , x_p w_p) \\ \end{array} \qquad & \qquad \begin{array}{c} \hbox {Power transformation} \\ \odot : \mathbb {R}\times \mathbb {S}^{p-1} \rightarrow \mathbb {S}^{p-1} \\ a \odot x:= C(x_1^a, x_2^a, \ldots , x_p^a) \\ \end{array} \end{array} \end{aligned}$$

Here, the *closure operator* $C: \mathbb {R}^p_{\ge 0} \rightarrow \mathbb {S}^{p-1}$ normalizes a *p*-dimensional, non-negative vector to the simplex $C(x):= x / \sum _{i=1}^p x_i$. Together with the dot-product

2 $$\begin{aligned} \langle x, w \rangle := \frac{1}{2 p} \sum _{i,j=1}^p \log \Bigl (\frac{x_i}{x_j}\Bigr ) \log \Bigl (\frac{w_i}{w_j}\Bigr ) \end{aligned}$$

the tuple $(\mathbb {S}^{p-1}, \oplus , \odot , \langle \cdot , \cdot \rangle )$ forms a finite-dimensional real Hilbert space^53^ allowing to transfer usual geometric notions such as lines and circles from Euclidean space to the simplex.

#### Coordinate representations

The *p* entries of a composition remain dependent via the unit sum constraint, leading to $\mathbb {S}^{p-1}$ having dimension $p-1$. To deal with this fact, different invertible log-based transformations have been proposed, for example the additive log ratio, centered log ratio^1^, and isometric log ratio^54^ transformations

3 $$\begin{aligned} {{\,\mathrm{\textrm{alr}}\,}}(x)= V_{\textrm{a}} \log (x) \in \mathbb {R}^{p-1},\quad {{\,\mathrm{\textrm{clr}}\,}}(x)= V_{\textrm{c}} \log (x) \in \mathbb {R}^{p},\quad {{\,\mathrm{\textrm{ilr}}\,}}(x)= V_{\textrm{i}} \log (x) \in \mathbb {R}^{p-1}, \end{aligned}$$

where the logarithm is applied element-wise and the matrices $V_{\textrm{a}}, V_{\textrm{i}} \in \mathbb {R}^{(p-1)\times p}$ and $V_{\textrm{c}} \in \mathbb {R}^{p\times p}$ are defined in Supplementary Material S2. While ${{\,\mathrm{\textrm{alr}}\,}}$ is a vector space isomorphism that preserves a one-to-one correspondence between all components except for one, which is chosen as a fixed reference point to reduce the dimensionality (we choose $x_p$, but any other component works), it is not an isometry, i.e., it does not preserve distances or scalar products. Both ${{\,\mathrm{\textrm{clr}}\,}}$ and ${{\,\mathrm{\textrm{ilr}}\,}}$ are also isometries, but ${{\,\mathrm{\textrm{clr}}\,}}$ only maps onto a subspace of $\mathbb {R}^p$, which often renders measure theoretic objects such as distributions degenerate. As an isometry between $\mathbb {S}^{p-1}$ and $\mathbb {R}^{p-1}$, ${{\,\mathrm{\textrm{ilr}}\,}}$ allows for an orthonormal coordinate representation of compositions. However, it is hard to assign meaning to the individual components of ${{\,\mathrm{\textrm{ilr}}\,}}(x)$, which all entangle a different subset of relative abundances in *x* leading to challenges for interpretability^55^. Therefore, ${{\,\mathrm{\textrm{alr}}\,}}$ remains a useful tool in statistical analyses where interpretability is required despite the lack of the isometric property.

#### Log-contrast estimation

The key advantage of such coordinate transformations is that they allow us to use regular multivariate data analysis methods (typically tailored to Euclidean space) for compositional data. For example, we can directly fit a linear model $y = \beta _0 + \beta ^T {{\,\mathrm{\textrm{ilr}}\,}}(x) + \epsilon$ on the ${{\,\mathrm{\textrm{ilr}}\,}}$ coordinates via ordinary least squares (OLS) regression. However, in real-world datasets, *p* is often a large number capturing “all possible components in a measurement”, leading to $p \gg n$ with each of the *n* measurements being sparse, i.e., a substantial fraction of *x* being zero. Moreover, in many (especially high-dimensional) situations only few components exert direct causal influence on the outcome. Both overparameterization $p\gg n$ as well as assuming sparse effects call for regularization. The problem with enforcing sparsity in a “linear-in-${{\,\mathrm{\textrm{ilr}}\,}}$” model is that a zero entry in $\beta$ does not correspond directly to a zero effect of the relative abundance of any single component. This motivates *log-contrast* estimation^56^ with a sparsity penalty^57–59^

4 $$\begin{aligned} \min _{\beta } \sum _{i=1}^n \mathscr {L}(x_i, y_i, \beta ) + \lambda \Vert \beta \Vert _1 \quad \text {s.t.}\, \sum _{i=1}^p \beta _i = 0 \,. \end{aligned}$$

In our examples, we focus mostly on continuous $y \in \mathbb {R}$ and the squared loss $\mathscr {L}(x, y, \beta ) = (y - \beta ^T \log (x))^2$. However, our framework also supports the Huber loss for robust log-contrast regression as well as an optional joint concomitant scale estimation for both losses^59,60^. Moreover, for classification tasks with $y \in \{0, 1\}$, we can directly use the squared Hinge loss (or a “Huberized” version thereof) for $\mathscr {L}$, see Supplementary Material S7 for details. These flexible estimation formulations respect the compositional nature of *x* while retaining the association between the entry $\beta _i$ and the relative abundance of the individual component $x_i$. Even though, due to the additional sum constraint, individual components of $\beta$ are still not—and can never be—entirely disentangled.

#### Logs and zeros

In the previous paragraphs, we introduced multiple log-based coordinate representations for compositions and at the same time claimed that measurements are often sparse in relevant settings. Since the logarithm is undefined for zero entries, a simple strategy is to add a small constant to all absolute counts, so called *pseudo-counts*^61,62^. These pseudo-counts are particularly popular in the microbiome and single-cell RNA literature where there are many more possible taxa/genes (up to tens of thousands) that occur in any given sample. Despite the simplicity of adding a constant pseudo-count, for example 0.5, recent work gives theoretical and empirical evidence for this approach^63^, which we also use here.

#### Summary statistics

Traditionally, interpretability issues around compositions have been circumvented by focusing on summary statistics instead of individual relative abundances. One of the key measures to describe ecological populations is *diversity*. Diversity captures the variation within a composition and is in this context often called $\alpha$-diversity. There is no unique definition of $\alpha$-diversity. Among the most common ones in the literature are *richness*, i.e. the number of non-zero entries denoted as $\Vert x\Vert _{0}$, *Shannon diversity* $-\sum _{j=1}^p x_j \log (x_j)$ and *Simpson diversity* $-\sum _{j=1}^p x_j^2$. Especially in the microbial context, there exist entire families of diversity measures taking into account species, functional, or phylogenetic similarities between taxa and tracing out continuous parametric profiles for varying sensitivity to highly-abundant taxa. See for example^64–66^ for an overview of the possibilities and choices of estimating $\alpha$-diversity in a specific application. While the popularity of $\alpha$-diversity for assessing the impact and health of microbial compositions^67^ seemingly renders it a natural choice for causal queries, we argue that such claims are misleading and void of a solid foundation.

### Methods for higher dimensional causes

In this section we develop methods to reason about the effects of hypothetical interventions on the relative abundance of individual components from observational data.

- *2SLS:* As the first baseline, we run 2SLS from Eq. (1) directly on $X \in \mathbb {S}^{p-1}$ ignoring its compositional nature.
- *Only LC* For completeness, as the second baseline, we run log-contrast (LC) estimation for the second stage only, thereby entirely ignoring confounding.
- $2SLS_{ILR}:$ 2SLS with ${{\,\mathrm{\textrm{ilr}}\,}}(X) \in \mathbb {R}^{p-1}$ as the treatment; since OLS minima do not depend on the chosen basis, parameter estimates for different log-transformations of *X* are related via fixed linear transformations. Hence, as long as no sparsity penalty is added, ${{\,\mathrm{\textrm{ilr}}\,}}$ and ${{\,\mathrm{\textrm{alr}}\,}}$ regression yield equivalent results. The isometric ${{\,\mathrm{\textrm{ilr}}\,}}$ coordinates are useful due to the consistency guarantees of 2SLS given that $\varvec{Z}^T {{\,\mathrm{\textrm{ilr}}\,}}(\varvec{X})$ has full rank. For interpretability, ${{\,\mathrm{\textrm{alr}}\,}}$ coordinates can be beneficial as individual coordinates correspond to individual components (given a reference). The respective coordinate transformations are given in Supplementary Material S2.
- $KIV_{ILR}:$ Following^45^ we replace OLS in 2SLS_ILR_ with kernel ridge regression in both stages to allow for non-linearities. Like 2SLS_ILR_, KIV_ILR_ cannot enforce sparsity in an interpretable fashion.
- *ILR+LC:* To account for sparsity, we use sparse log-contrast estimation (see Eq. (4)) for the second stage, while retaining OLS to ${{\,\mathrm{\textrm{ilr}}\,}}$ coordinates for the first stage. Log-contrast estimation conserves interpretability in that the estimated parameters correspond directly to the effects of individual relative abundances.
- *DIR+LC:* Finally, we circumvent log-transformations entirely and deploy regression methods that naturally work on compositional data in both stages. For the first stage, we use a Dirichlet distribution—a common choice for modeling compositional data—where $X\mid Z \sim \textrm{Dirichlet}(\alpha _1(Z), \ldots , \alpha _p(Z))$ with density $B(\alpha _1, \ldots , \alpha _p)^{-1} \prod _{j=1}^{p}x_j^{\alpha _j - 1}$ where we drop the dependence of $\alpha = (\alpha _1,\ldots ,\alpha _p) \in \mathbb {R}^p$ on *Z* for simplicity. With the mean of the Dirichlet distribution given by $\nicefrac {\alpha }{\sum _{j=1}^p \alpha _j}$, we account for the *Z*-dependence via $\log (\alpha _j(Z_i)) = \omega _{0,j} + \omega _j^T Z_j$. We then estimate the newly introduced parameters $\omega _{0,j} \in \mathbb {R}$ and $\omega _j \in \mathbb {R}^q$ via maximum likelihood estimation with $\ell _1$ regularization. For the second stage we again resort to sparse log-contrast estimation. If the non-linear first stage is misspecified, the “forbidden regression” bias may distort effect estimates of this approach. This is contrasted by Dirichlet regression potentially resulting in a better fit of the data than linearly modeling log-transformations.

We highlight that only ILR+LC and DIR+LC accommodate all relevant requirements: (i) unobserved confounding, (ii) compositional treatments, (iii) sparse effects, and (iv) interpretable estimates. Additionally, we wish to emphasize the concept of “forbidden regression” as discussed in^37^. This issue arises specifically when a nonlinear first stage is improperly combined with a subsequent regression stage. With regard to the proposed methods, this issue will only affect *DIR + LC*, which we will further assess in the simulations. Both *2SLS* and *Only LC* are inherently consistent. Meanwhile, ILR+LC, 2SLS_ILR_, and KIV_ILR_ derive their consistency from linear techniques resp.^45^, as the log-transformation is itself linear. Therefore, the linear transformation ensures that there is no “forbidden” non-linearity introduced in the first stage regression step.

## Simulation studies

### Data generation

**Fig. 3:**
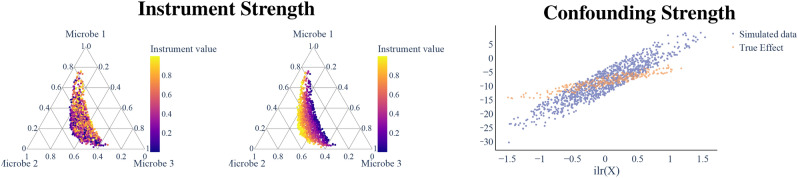
Visualization of a Setting A ($p=3, q=2$): The left panel shows both a weak (left) and a strong (right) instrument, i.e. with the *instrument value* *Z* either barely influencing the the composition of the microbiome (left) or strongly impacting the composition of the microbiome (right). The right panel shows a discrepancy between the true causal effect and the observed effect which stems from a confounding factor.

For the evaluation of our methods we require the ground truth causal effect to be known. Since unobserved confounders (and thus counterfactuals) are never observed in practice (by definition), this can only be achieved via synthetic data. We simulate data (in two different settings) to maintain control over ground truth effects, confounding strength, potential misspecification, and the strength of instruments (see Fig. 3).

- *Setting A:* The first setting is
  5 $$\begin{aligned} & Z_j \sim \textrm{Unif}(0, 1),\qquad U \sim \mathscr {N}(\mu _c, 1), \nonumber \\ & {{\,\mathrm{\textrm{ilr}}\,}}(X) = \gamma _0 + \gamma ^T Z + U c_X,\quad Y = \beta _0 + \beta ^T {{\,\mathrm{\textrm{ilr}}\,}}(X) + U c_Y, \end{aligned}$$
  where we model ${{\,\mathrm{\textrm{ilr}}\,}}(X) \in \mathbb {R}^{p-1}$ directly and $\mu _c, c_Y \in \mathbb {R}$, $\gamma _0, c_X \in \mathbb {R}^{p-1}$, $\gamma \in \mathbb {R}^{q \times (p-1)}$ are fixed up front. Our goal is to estimate the causal parameters $\beta \in \mathbb {R}^{p-1}$ and the intercept $\beta _0 \in \mathbb {R}$. This setting satisfies the standard 2SLS assumptions (linear, additive noise) and all our linear methods are thus *wellspecified*. To explore effects of *misspecification*, we also consider the same setting only replacing (using $\varvec{1} = (1, \ldots , 1)$)
  6 $$\begin{aligned} Y = \beta _0 + \frac{1}{100} \varvec{1}^T({{\,\mathrm{\textrm{ilr}}\,}}(X) + 1)^2 + 10 \cdot \varvec{1}^T \sin ({{\,\mathrm{\textrm{ilr}}\,}}(X)) + c_Y U. \end{aligned}$$
- *Setting B:* We consider a sparse effect model for $X \in \mathbb {S}^{p-1}$ which is more realistic for higher-dimensional compositions. Note that some parameter dimensions are different, i.e., the same symbols have different meanings in the settings A and B. With $\mu = \gamma _0 + \gamma ^T Z$ for fixed $\gamma _0 \in \mathbb {R}^p$, $\gamma \in \mathbb {R}^{q \times p}$ we use
  7 $$\begin{aligned} & Z_j \sim \textrm{Unif}({Z_{\min }}, {Z_{\max }} ),\qquad U \sim \textrm{Unif}({U_{\min }}, {U_{\max }}), \nonumber \\ & X \sim C \bigl (\textrm{ZINB}(\mu , \Sigma , \theta , \eta ) \bigr ) \oplus (U \odot \Omega _C), \nonumber \\ & Y = \beta _0 + \beta ^T \log (X) + c_Y^T \log (U \odot \Omega _C). \end{aligned}$$
  The treatment *X* is assumed to follow a zero-inflated negative binomial (ZINB) distribution^68^, commonly used for modelling count data with excess zeros^69^. Here, $\eta \in (0, 1)$ is the probability of zero entries, $\Sigma \in \mathbb {R}^{p\times p}$ is the covariance matrix, and $\theta \in \mathbb {R}$ the shape parameter. The confounder $U \in [U_{\min }, U_{\max }]$ perturbs this base composition in the direction of another fixed composition $\Omega _C \in \mathbb {S}^{p-1}$ scaled by *U*. In simplex geometry $x_0 \oplus (U \odot x_1)$ corresponds to a line starting at $x_0$ and moving along $x_1$ by a fraction *U*. A linear combination of the log-transformed perturbation enters *Y* additively with weights $c_Y \in \mathbb {R}^p$ controlling confounding strength. All other parameter choices are given in Supplementary Material S6. This setting is linear in how *Z* enters $\mu$ and how *U* enters *X* and *Y* in the simplex geometry. All our two-stage models are (intentionally) misspecified in the first stage for setting B.

The precise choices of all parameters for the different empirical evaluations are described in the appendix (Supplementary Material S6). All relevant code is available at https://github.com/EAiler/causal-compositions.

### Metrics and evaluation

Appropriate evaluation metrics are key for cause-effect estimation tasks. We aim at capturing the average causal effect (under interventions) and the causal parameters when warranted by modeling assumptions. When the true effect is linear in $\log (X)$, we can compare the estimated causal parameters $\hat{\beta }$ from 2SLS_ILR_, ILR+LC, and DIR+LC with the ground truth $\beta$ directly. In these linear settings, we report causal effects of individual relative abundances $X_j$ on the outcome *Y* via the mean squared difference ($\beta$**-MSE**) between the true and estimated parameters $\beta$ and $\hat{\beta }$. Moreover, we also report the number of falsely predicted non-zero entries (**FNZ**) and falsely predicted zero entries (**FZ**), which are most informative in sparse settings and metrics of key interest to biostatisticians.

In the general case, where a measure for identification of the interventional distribution $P(Y \mid do(X))$ is not straightforward to evaluate, we focus on the *out of sample error* (**OOS MSE**): For the true causal effect we first draw an i.i.d. sample $\{x_i\}_{i=1}^{m}$ from the data generating distribution (that are not in the training set, i.e., out of sample) and compute ${{\,\mathrm{\mathbb {E}}\,}}_{U}[f(x_i, U)]$ for the known *f*(*X*, *U*), the expected *Y* under intervention $do(x_i)$. We use $m=250$ for all experiments. OOS MSE is then the mean square difference to our second-stage predictions $\hat{f}(x_i)$ on these out of sample $x_i$. Because in real observational data we do not have access to $P(Y \mid do(X))$ (but only the conditional distribution $P(Y \mid X)$, we can not evaluate OOS MSE in real-world observational data.

We run each method for 50 random seeds in setting A (Eq. (5)), and 20 random seeds in setting B (Eq. (7)). In result tables, we report mean and standard error over these runs. The sample size is $n=1000$ in the low-dimensional case ($p=3$) and $n=10,\!000$ in the higher-dimensional cases ($p=30$, $p=250$). Additionally, we report results for an overparameterized setting with $n=100$ and $p=250$. Supplementary Material S6 and S8 contain further explanations and more detailed results. Note, that since ${{\,\mathrm{\textrm{alr}}\,}}$ coordinates for *X* yield equivalent optimization minima as ILR+LC, we only report results from ILR+LC. All numbers match precisely for ALR+LC in our empirical evaluation.

**Table 1:** Results for setting A (fully linear in ${{\,\mathrm{\textrm{ilr}}\,}}(X)$). Bold values indicate the lowest OOS MSE resp. β-MSE in the corresponding dimension scenario.

		Setting A, Equation (5)			
Dim.	Method	OOS MSE	$\beta$-MSE	FZ	FNZ
$p=3$ $q=2$	DIR+LC	$0.58$ $\pm 0.08$	$1.6$ $\pm 0.17$	0.0	0.0
	ILR+LC$^{\dagger }$	$\varvec{0.37}$ $\pm 0.07$	$\mathbf {1.1}$ $\pm 0.15$	0.0	0.0
	KIV_ILR_	$\varvec{0.37}$ $\pm 0.07$	–	–	–
	Only LC	$15.03$ $\pm 0.20$	$32.6$ $\pm 0.14$	0.0	0.0
	2SLS	$>200$	$>5$k	0.0	0.0
$p=30$ $q=10$	ILR+LC	$\varvec{0.42}$ $\pm 0.08$	$\varvec{0.22}$ $\pm 0.01$	0.0	12.0
	KIV_ILR_	$240.6$ $\pm 35.7$	–	–	–
	Only LC	$24.4$ $\pm 0.37$	$1.9$ $\pm 0.00$	0.0	12.3
$p=250$ $q=10$	ILR+LC	$\varvec{0.67}$ $\pm 0.14$	$\varvec{0.22}$ $\pm 0.02$	0.0	0.0
	KIV_ILR_	$5060.5$ $\pm 1196.2$	–	–	–
	Only LC	$30.8$ $\pm 0.48$	$143.3$ $\pm 0.27$	3.0	1.0

$^{\dagger }$ Identical to 2SLS_ILR_ in low-dimensional setting without sparsity

### Results for low-dimensional compositions

We first consider settings A and B with $p=3$ and $q=2$. The top section of Tables 1 and 2 shows our metrics for all methods. First, effect estimates are far off when ignoring the compositional nature (2SLS) or the confounding (Only LC) as expected. Also, recent non-linear IV methods such as^47,70,71^ could not overcome the issues of 2SLS in this setting. Without sparsity in the second stage, 2SLS_ILR_ and ILR+LC yield equivalent estimates in this low-dimensional linear setting—we only report ILR+LC. ILR+LC (and equivalent methods) succeed in cause-effect estimation under unobserved confounding: they recover the true causal parameters with high precision on average (low $\beta$-MSE) and thus achieve low OOS MSE. While DIR+LC performs reasonably well in setting A, setting B surfaces that despite being a seemingly plausible approach with powerful regression techniques, DIR+LC suffers substantially under a misspecified first-stage.

**Table 2:** Results for setting B (first stage ZINB with sparse effects in higher dimensions), where all our two-stage methods are (intentionally) misspecified in the first stage. Bold values indicate the lowest OOS MSE resp. β-MSE in the corresponding dimension scenario.

		Setting B, Equation (7)			
Dim.	Method	OOS MSE	$\beta$-MSE	FZ	FNZ
$p=3$ $q=2$	DIR+LC	$>10$k	$>2$k	0.0	0.0
	ILR+LC$^{\dagger }$	$\varvec{20.3}$ $\pm 4.85$	$\varvec{9.9}$ $\pm 3.3$	0.0	0.0
	KIV_ILR_	$\varvec{19.2}$ $\pm 4.42$	–	–	–
	Only LC	$269.0$ $\pm 6.85$	$129.8$ $\pm 2.13$	0.0	0.0
	2SLS	$>15$k	$>300$k	0.0	0.0
$p=30$ $q=10$	ILR+LC	$\varvec{120.4}$ $\pm 25.1$	$\varvec{37.0}$ $\pm 15.1$	0.0	13.3
	KIV_ILR_	$287.2$ $\pm 19.1$	–	–	–
	Only LC	$3863.8$ $\pm 166.3$	$458.8$ $\pm 12.2$	3.4	15.8
$p=250$ $q=10$	ILR+LC	$\varvec{99.1}$ $\pm 7.9$	$\varvec{24.4}$ $\pm 4.30$	0.13	0.39
	KIV_ILR_	$622.8$ $\pm 30.1$	–	–	–
	Only LC	$3366.0$ $\pm 166.3$	$498.3$ $\pm 17.2$	6.9	1.9

$^{\dagger }$ Identical to 2SLS_ILR_ in low-dimensional setting without sparsity

**Fig. 4:**
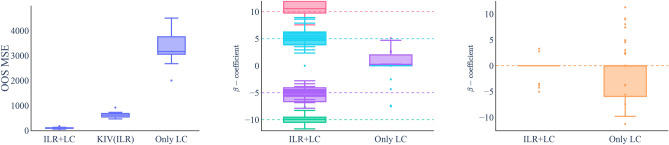
Boxplots of the results for setting B in Table 2 with $p=250, q=10$. We show OOS MSE (left), recovery of non-zero $\beta$ coefficients (middle), and recovery of zero $\beta$ coefficients.

### Results for high-dimensional compositions

We now consider the challenging cases $p=30$ and $p=250$ with $q=10$ and sparse ground truth $\beta$ for settings A and B (8 non-zeros: 3 times $-5$ and 5 and once $-10$ and 10) in the bottom sections of Tables 1 and 2. ILR+LC deals well with sparsity: unlike Only LC, it identifies non-zero parameters perfectly ($\textrm{FZ}=0$) and rarely predicts false non-zeros. It also identifies the true $\beta$ and accordingly predicts interventional effects (OOS MSE) well. DIR+LC and 2SLS_ILR_ fail entirely in these settings because the optimization does not converge. While we could get KIV_ILR_ to return a solution, tuning the kernel hyperparameters for high-dimensional ${{\,\mathrm{\textrm{ilr}}\,}}$ coordinates becomes increasingly challenging, which is reflected in poor OOS MSE. In Fig. 4 we show detailed results for the most challenging setting (setting B with $p=250$ and $q=10$) including the OOS MSE (left), recovery of individual non-zero coefficients (middle), and recovery of zero coefficients (right). Analogous plots for all other settings can be found in Supplementary Material S8.

**Fig. 5:**
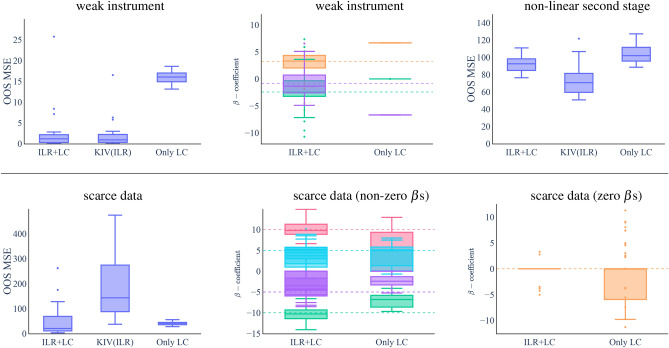
We display OOS MSE and $\beta$-MSE (for non-zero coefficients and where applicable) for our robustness checks. All results and further visualizations are in Supplementary Material S8.

### Robustness checks

Due to the inherent entanglement via the unit sum constraint, analyses involving compositional data are generically hard to interpret. Causal analyses involving compositions in the instrumental variable setting are further challenged by potential violations of assumptions such as weak instruments or misspecification. We assess the sensitivity of our proposed methodology to such potential pitfalls in the following scenarios.

#### Weak instruments

“Strong instruments” are a prerequisite for successful two-stage estimation in the instrumental variable setting and one of the key discussion points in real-world applications of IV. Nevertheless, how to measure instrument validity is not unambiguously clarified, there is no clear definition on which instruments are weak and which instruments are strong. The categories rely on heuristics and empirically derived best practices. In the linear setting, instrument strength for $p=1$ can be approximated via a first-stage F-statistic with a value greater than 10 generally being considered sufficient to avoid weak instrument bias in 2SLS^72^. For $p>1$, measuring instrument strength is more challenging even in the linear case^73^. Therefore, we report first-stage F-statistics for each dimension of *X* as a proxy for instrument strength.

When instruments are weak, the estimation bias can theoretically become arbitrarily large (even in the limit of infinite data). To assess the sensitivity of our methods to weak instrument bias, we re-analyze setting A ($p=3$ and $q=2$) only changing the dependence of *X* and *Z* to be weak with first-stage F-statistic values of 6.9 and 4.7 for the two components of ${{\,\mathrm{\textrm{ilr}}\,}}(X)$. In the linear setting, we can directly control instrument strength via $\alpha$ (see Eq. (5)).

The first row in Table 3 summarizes our results for weak instruments: the two-stage methods have a substantially higher variation in their estimates, both for OOS MSE and $\hat{\beta }$ compared to the strong instrument setting in Tables 1 and 2. As the second stage has not changed, Only LC performs equally bad. Notably, while the wellspecified two-stage methods ILR+LC and 2SLS_ILR_ seem to do worse than Only LC, the large OOS MSE and $\beta$-MSE are mostly due to outliers. Fig. 5 shows that the range of $\beta$ estimates still cover the true values for ILR+LC and 2SLS_ILR_, while Only LC is systematically off with low variance (confidently wrong). DIR+LC now not only suffers from the misspecified first stage but also the weak instrument resulting in virtually useless estimates. The surprisingly good performance of KIV_ILR_ in this specific setting is unexpected and cannot be consistently reproduced over different weak instrument scenarios: the performance is highly volatile and often worse than ILR+LC. Therefore, despite the good performance for these specific parameters, we find that more flexible methods are also affected heavily by weak instruments. In general, while two-stage estimates generally cannot be broadly trusted when instruments are weak, reverting to Only LC is potentially even more detrimental because the estimated coefficients are systematically off.

#### Non-linear second stage

Well-specification is typically impossible to ascertain in practice and most real-world examples are likely not perfectly linear even when the linearity assumption can be defended. Therefore, we introduce a non-linear *f* for setting A with $p=3$ and $q=2$ (Equation (6)), resulting in a misspecified second stage for all our methods except KIV_ILR_, which can in principle capture non-linearities. Note that $\beta$ cannot be interpreted directly as causal parameters when the true causal effect depends non-linearly on ${{\,\mathrm{\textrm{ilr}}\,}}(X)$. The results in the second row of Table 3 show that DIR+LC (doubly misspecified) and 2SLS (ignoring compositionality) again fail. Moreover, in this non-linear scenario KIV_ILR_ beats ILR+LC (both still outperforming Only LC) and we expect the difference to grow as the non-linearity of *f* increases.

#### Scarce data

Finally, we return to the original setting A ($p=250$, $q=10$, linear in both stages), but mimic a scarce data scenario with $n=100$. The third row in Table 3 clearly highlights again how the lack of regularization becomes problematic for 2SLS_ILR_ and KIV_ILR_. Compared to the larger dataset, also our regularized two-stage methods naturally exhibit higher variation in their estimates. Notably, Only LC appears to compare favorably to ILR+LC in OOS MSE, but $\beta$-MSE surfaces its failure to accurately recover causal parameters. We thus conclude that despite increased variability, the ILR+LC is still better equipped to recover $\beta$ in the small data regime (see Fig. 5).

**Table 3:** Results for various robustness checks. Bold values indicate the lowest OOS MSE resp. β-MSE in the relevant dimension scenario.

Scenario	Method	OOS MSE	$\beta$-MSE
Weak Instruments	DIR+LC	$7.1$ $\pm 2.6$	$43.2$ $\pm 10.3$
	ILR+LC$^{\dagger }$	$3.6$ $\pm 1.3$	$26.0$ $\pm 5.6$
	KIV_ILR_	$\varvec{2.7}$ $\pm 0.9$	–
	Only LC	$15.9$ $\pm 0.20$	$52.2$ $\pm 0.18$
	2SLS	$>100$	$>5$k
Non-Linearity	DIR+LC	${135.6}$ $\pm 6.34$	–
	ILR+LC$^{\dagger }$	${92.0}$ $\pm 1.2$	–
	KIV_ILR_	$\varvec{73.4}$ $\pm 2.28$	–
	Only LC	$104.1$ $\pm 1.43$	–
	2SLS	$>300$	–
Scarce Data	ILR+LC	$\varvec{45.1}$ $\pm 7.90$	$\varvec{72.8}$ $\pm 8.3$
	KIV_ILR_	$290.8$ $\pm 62.5$	–
	Only LC	$\varvec{40.5}$ $\pm 1.00$	$196.4$ $\pm 8.7$
	2SLS_ILR_	$>10$k	$>2 \cdot 10^{24}$

$^{\dagger }$ Identical to 2SLS_ILR_ in low-dimensional setting without sparsity

## Case study on murine sub-therapeutic antibiotic treatment

We consider the mouse dataset described by^74^ and analyzed in^30^ using causal mediation. A total of 57 newborn mice were assigned randomly to a sub-therapeutic antibiotic treatment (STAT) during their early stages of development. Sub-therapeutic antibiotic treatment means that the administered doses of antibiotics are too small to be detectable in the mice’ bloodstream. There were 35 mice in the treatment group and 22 mice in the control group. After 21 days, the gut microbiome composition of each mouse was recorded. We are interested in the causal effect of the gut microbiome composition on body weight $Y \in \mathbb {R}$ of the mice (at sacrifice).

We assume a valid instrument due to the following characteristics in the data generation: The random assignment of the antibiotic treatment ensures independence of potential confounders such as genetic factors ($Z {{\,\mathrm{\perp \!\!\!\perp }\,}}U$). The sub-therapeutic dose implies that antibiotics can not be detected in the mice’ blood, providing reason to assume no effect of the antibiotics on the weight other than through its effect on the gut microbiome ($Z {{\,\mathrm{\perp \!\!\!\perp }\,}}Y \mid \{U, X\}$).

Finally, we observe empirically, that there are statistically significant differences of microbiome compositions between the treatment and control groups ($Z{{\,\mathrm{\not \perp \!\!\!\perp }\,}}X$) based on the first stage F-statistic. Thus, the sub-therapeutic antibiotic treatment is a good candidate for an instrument $Z \in \{0, 1\}$ in estimating the effect $X \rightarrow Y$. Note, however, that this work is focused on methods rather than novel biological insights as more scrutiny of the IV assumptions would be required for substantive biological claims.

**Fig. 6:**
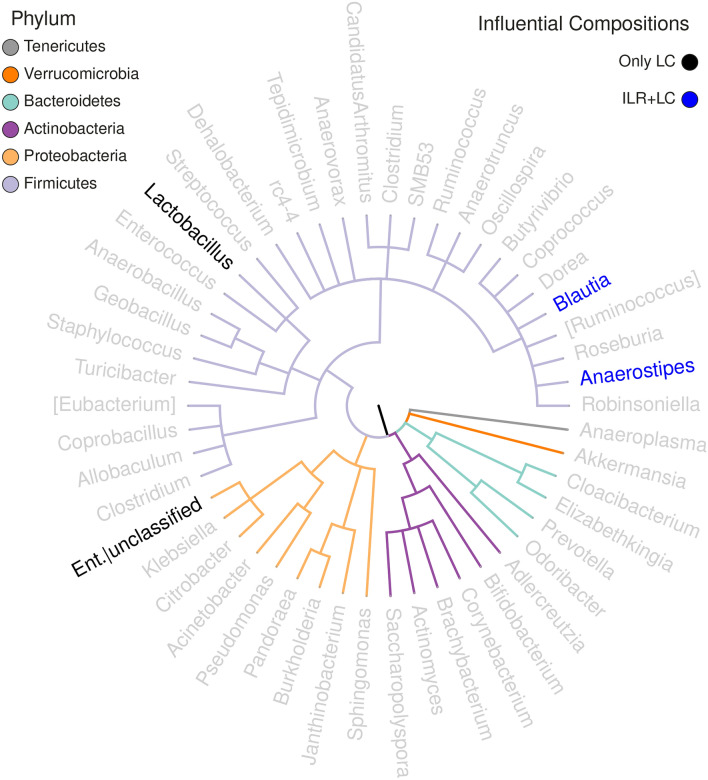
Taxonomic tree of the microbiome data at genus level. The influential log-ratios for both Only Second LC and ILR+LC are highlighted in black and blue, respectively.

Figure 6 highlights the two most influential microbes on the genus level for our two-stage ILR+LC estimator and to the non-causal baseline Only LC, respectively. In the causal setting, we estimate the log-ratio of Blautia to Anaerostipes to be most influential for weight gain whereas standard log-contrast regression deems the ratio of an unclassified Enterobacteria genus to Lactobacillus to be the most predictive genus pair. This discrepancy suggests that the second stage might be subject to confounding. However, the mediation analysis on the same dataset in^30^ posits a negative mediation effect of Lactobacillus on weight gain, consistent with the non-causal baseline model. This highlights the fact that different causal models provide alternative interpretation of the data that can only resolved by follow-up biological experiments.

Finally, we also assessed the influence of taxonomic aggregation levels and different loss functions as well as different values of zero counts on the results (see Supplementary Material S5). We observed that our causal model is robust to the choice of the loss function in terms of selected taxa whereas the baseline model found loss-function depdendent sets of predictive taxa (see Supplementary Material Figure S2).

## Discussion

In this work, we developed and analyzed methods for cause-effect estimation with compositional causes under unobserved confounding in instrumental variable settings. First, we succinctly expose that the common portrayal of summary statistics as a decisive (rather than merely descriptive) description of compositions is misguided. Instead, we advocate for causal effects to be estimated from the entire composition vector directly to establish meaningful and interpretable causal links. As a result, analysts cannot tap into a collection of well established cause-effect estimation tools for scalar data, but are instead faced with a large number of possible components (calling for sparsity-enforcing methods) and typically have to deal with unobserved confounding. Given the potentially profound impact of microbiome or single cell RNA data on advancing human health or of species abundances on global health, it is of vital importance that we face these challenges and develop interpretable methods to obtain causal insights from compositional data.

To this end, we carefully developed and assessed the effectiveness of various methods to not only reliably recover causal effects (OOS MSE), but also yield *interpretable and sparse* effect estimates for individual abundances ($\beta$-MSE, FZ, FNZ) whenever applicable. We also put special emphasis on how IV assumptions (misspecification, weak instrument bias) interact with compositionality. Our extensive empirical results for different two-stage methods highlight that accounting for the compositional nature as well as confounding is not optional. The overall failure of DIR+LC shows that not any seemingly suitable compositional technique can be trusted to yield reliable estimates in a manual two-stage procedure—careful analysis is needed. We have identified ILR+LC, to work reliably in wellspecified sparse and non-sparse settings as well as being relatively robust to first- and (small) second-stage misspecifications (i.e., non-linearities) and scarce data. It also yields interpretable estimates for individual components. When interpretability is not required or second-stage non-linearities are strong, KIV_ILR_ can still perform well under these relaxed assumptions albeit being challenging to tune for large *p* and unable to incorporate sparsity. As expected, valid instruments are required for all our two-stage methods. Taken together, our results on the efficacy and robustness of our methods in simulation and on real microbiome data provide first recommendations for practitioners to fully integrate compositional data into cause-effect estimation.

## Supplementary Information

Supplementary Information.

## Data Availability

All relevant code and data to reproduce the experiments, results and visualizations is available at https://github.com/EAiler/causal-compositions. The folder “/input/data” includes the data files (.Rdata) as well as the preprocessing steps (.Rmd). The folder “/notebooks” includes all relevant steps to understand the analysis steps and the visualizations, while the folder “/src” includes relevant background code.
