# Supplementary material for: Instrumental variable estimation for compositional treatments
Source: Sci Rep. 2025 Feb 12;15:5158. doi: 10.1038/s41598-025-89204-9 (PMC11814326; doi:10.1038/s41598-025-89204-9)
Supplement: Supplementary file 1 — Supplementary Information. [file 41598_2025_89204_MOESM1_ESM.pdf]

S1 Supplementary Material

The supplementary material contains details on compositional data transformations (Supplementary Material S2) and the applied instrumental variables methods (Supplementary Material S3) as well as a list of the packages that have been used in the implementation (Supplementary Material S4). Further, the supplementary material provides additional results for the real data example of [1] (Supplementary Material S5). Moreover for the synthetic settings it holds a detailed description of the data generation (Supplementary Material S6), the parameter settings for the training of the methods (Supplementary Material S7) as well as additional results and visualizations (Supplementary Material S8).

S2 Compositional Data Transformations

Given a compositional vector $x \in \mathbb{S}^{p-1}$, the definitions of the log-transformations are given by the additive log-ratio transformation

$$\operatorname{alr}(x) := \left(\log \frac{x_1}{x_p}, \dots, \log \frac{x_{p-1}}{x_p}\right) = \tilde{x}_{\text{alr}} = \log(x) \cdot \begin{bmatrix} 1 & 0 & \cdots & 0 \\ 0 & 1 & \cdots & 0 \\ \vdots & \vdots & \ddots & \vdots \\ 0 & 0 & \cdots & 1 \\ -1 & -1 & \cdots & -1 \end{bmatrix} \tag{1}$$

with inverse

$$\operatorname{alr}^{-1}(\tilde{x}) = C(\exp([\tilde{x}, 0])), \tag{2}$$

the centered log-ratio transformation

$$\operatorname{clr}(x) := \left(\log \frac{x_1}{g(x)}, \dots, \log \frac{x_p}{g(x)}\right) = \tilde{x}_{\text{clr}} = \frac{\log(x)}{D} \cdot \begin{bmatrix} D-1 & -1 & \cdots & -1 \\ -1 & D-1 & \cdots & -1 \\ \vdots & \vdots & \ddots & \vdots \\ -1 & -1 & \cdots & D-1 \end{bmatrix} \tag{3}$$

with $g(x) := \sqrt[p]{x_1 \cdot \dots \cdot x_p}$ and inverse

$$\operatorname{clr}^{-1}(\tilde{x}) = C(\exp([\tilde{x}])), \tag{4}$$

and the isometric log-ratio transformation

$$\operatorname{ilr}_V(x) = \tilde{x}_{\text{ilr}} = \operatorname{clr}(x) \cdot V \tag{5}$$

for a matrix $V \in \mathbb{R}^{p \times p-1}$ such that $V^T V = \mathbb{I}_{p-1}$ providing an orthonormal basis of $\mathbb{R}^{p-1}$ with inverse

$$\operatorname{ilr}_V^{-1}(\tilde{x}) = C(\exp([\tilde{x} V^T])). \tag{6}$$

For the ilr transformation, a typical choice for V^T is the so-called Helmert matrix with the first row removed (see for example `http://scikit-bio.org/docs/0.4.1/generated/generated/skbio.stats.composition.ilr.html`).

Additionally let us illustrate how log transformations impact the coordinates. The ternary plot in Fig. S1 shows different levels of Shannon Diversity for a composition in $\mathbb{S}^3$ and its different log-transformations in $\mathbb{R}^2$ resp. $\mathbb{R}^3$.

S3 IV Methods

We consider three different approaches that gradually relax some of the common IV assumptions. In particular, the restrictions on the function space of f are gradually relaxed in the different settings.

The Two-Stage Least Squares algorithm (2SLS) consists of two sequential OLS regressions [2]. 2SLS is one of the most prominent approaches. It allows for unobserved confounding while still putting linear restrictions on the function space of f and assuming additive noise:

$$Y = \beta X + \varepsilon_Y \tag{7}$$

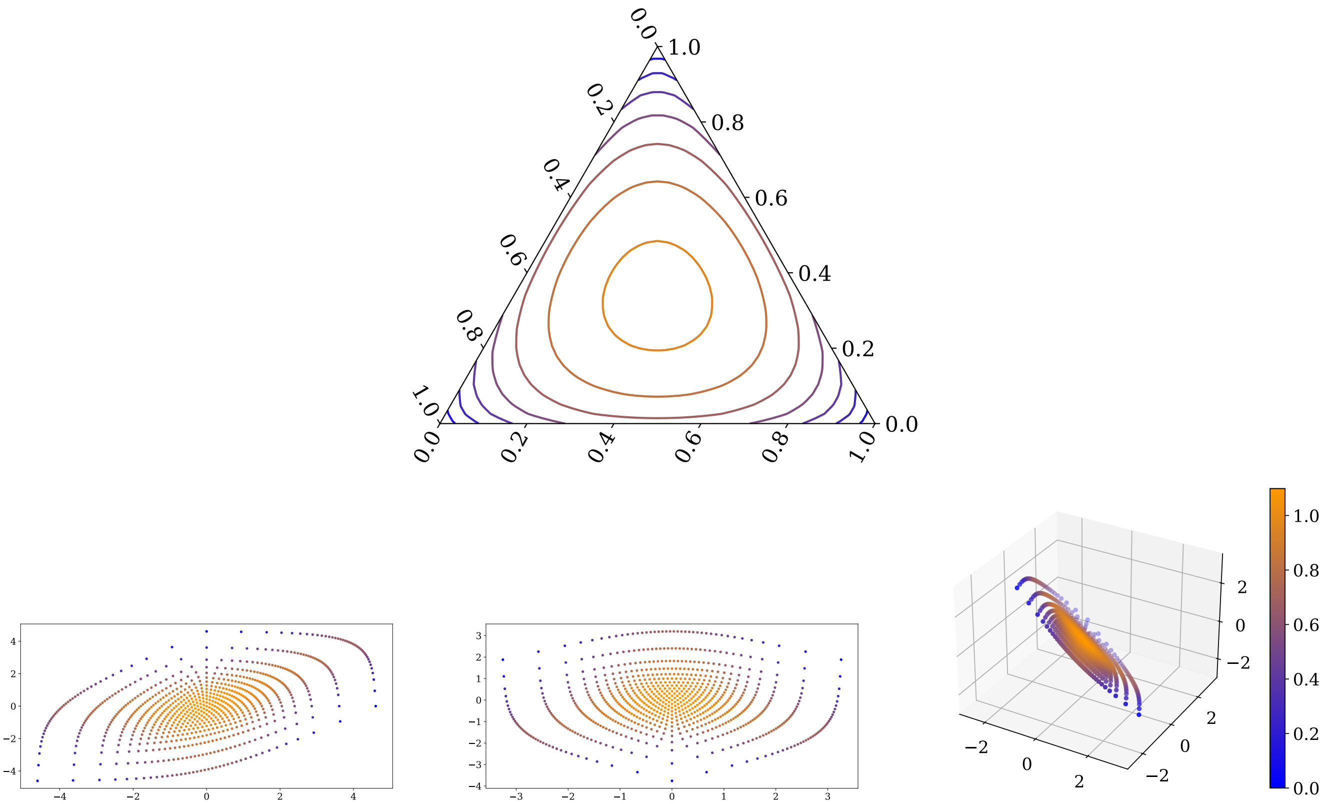

alr**-transformation** ilr**-transformation** clr**-transformation**

Figure S1. The ternary plot shows different levels of Shannon diversity for a microbial composition in $\mathbb{S}^3$. The different log-transformations convert the composition into $\mathbb{R}^2$ for ilr and alr-transformations resp. $\mathbb{R}^3$ for the clr-transformation.

First, 2SLS fits a regression model based on Z to predict X. The second stage uses the estimated $\hat{X}$ to predict Y. This results in the following estimator for β:

$$\hat{\beta} = \left(X^T P_Z X\right)^{-1} \left(X^T P_Z y\right) \tag{8}$$

with $P_Z = Z(Z^T Z)^{-1} Z^T$.

If $p = q$, the estimator reduces to the following form:

$$\hat{\beta} = \left(Z^T X\right)^{-1} \left(Z^T y\right) \tag{9}$$

[3] relax the assumption of the linear setting in 2SLS towards a non-parametric generalization of the causal effect by applying kernel ridge regression (KIV).

$$Y = f(X) + \varepsilon_Y, \tag{10}$$

for a potentially non-linear f, maintaining the additive noise assumption for point-identifiability.

The OLS regressions are replaced by kernel ridge regressions and thus model the relationship of Z, X and Y by non-linear functions in reproducing kernel Hilbert spaces (RKHSs). This method still requires additive noise models to produce consistent results. Following the arguments in [3], this gives us a closed form solution for f:

$$W = K_{XX}(K_{ZZ} + n\lambda Id)^{-1} K_{Z\tilde{Z}} \tag{11}$$

$$\hat{\alpha} = (WW^T + m\xi K_{XX})^{-1} W\tilde{y}, \tag{12}$$

$$\hat{f}_{\xi}^{m}(x) = (\hat{\alpha})^T K_{Xx} \tag{13}$$

In the next step, we drop the assumption of additive noise, i.e., allowing $f(X,U)$ to depend on the treatment X and any (potentially high-dimensional) confounder U in arbitrary ways (also non-linearly). This implies that the effect is only partially identifiable, i.e., we can only put lower and upper bounds on $\mathbb{E}[Y \mid do(x)]$. The authors in [4] employ the response function framework to minimize (maximize) the average causal effect over all causal models that satisfy the structural IV assumptions and simultaneously match the observed data to find the lower (upper) bound. We refer the reader to the original paper for the details [4].

S4 Package References

Here, we briefly outline the software used in our empirical evaluation. Please note that the code and the requirements are all available at https://github.com/EAiler/causal-compositions.

Python Packages

We use the following Python [5] packages: Plotly [6], Numpy [7], Scipy [8], scikit-learn [9], scikit-bio [10], rpy2 [11], Matplotlib [12], Statsmodels [13], Pandas [14], Jax [15], Dirichlet [16], c-Lasso [17].

R Packages

We use the following R [18] packages: SpiecEasi [19], vegan [20], Compositional [21] and metaSparSim [22].

S5 Case study on murine sub-therapeutic antibiotic treatment

In this part we turn to the analysis of the microbiome instead of the summary statistic as the cause. This is a more detailed examination of the murine sub-therapeutic antibiotic treatment data given in the main part. We provide results on higher aggregation levels, i.e., the taxonomic ranks 'Order' and 'Family', respectively. Moreover, we discretize the weight outcome Y and replace the squared loss of the log-contrast regression by a Hinge loss (see Supplementary Material S7).

—*Further Results on different Aggregation Levels:* Naturally, for real data, we do not have ground truth labels available. However, the importance of being able to draw causal and actionable conclusions becomes apparent. In the main part we provide the results for the naive regression and the two-stage method ILR+LC on genus level. The methods did not agree on the influential log-ratios, thus suggesting that Only LC might be subject to confounding. This result also holds true on family level. However, on order level both methods detect one common log-ratio (see Figure S2).

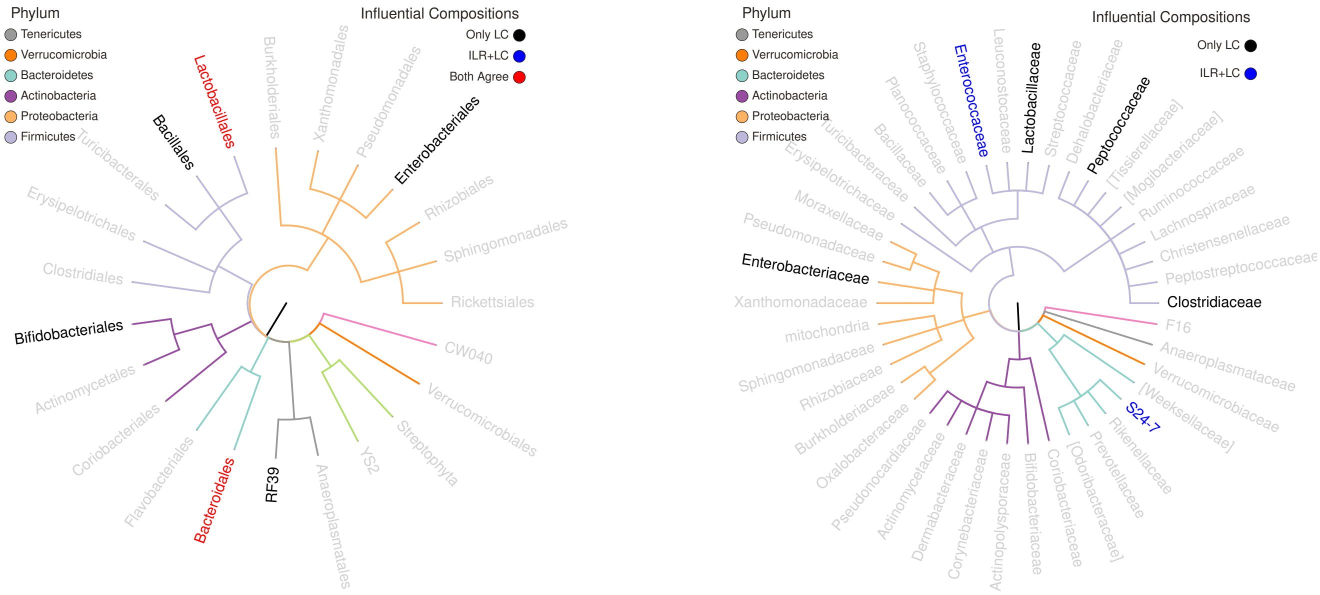

Figure S2. Influential Compositions on Order Level (left) and Family Level (right): On order level, both methods agree on one influential log ratio. For the family level, there is a divide between the two-stage method and the naive regression. This could suggest that Only LC is subject to confounding on the corresponding aggregation level.

—*Categorical/Binary Outcome:* In order to provide a more complete picture of the loss possibilities, we include results for a categorical/binary Y. Originally, the real data includes weight measured in gram. To create a binary outcome, we split the data by the mean of the outcome Y thus artificially generating an "underweight" population of 29 mice and an "overweight" population of 28 mice. Again, we show the influential log-ratios for the naive regression Only LC and ILR+LC (see Figure S3). While for ILR+LC the influential log-ratios stay the same for binary and continuous outcome, for the naive regression they are not entirely consistent.

—*Different Levels of Pseudo Counts:* The choice of pseudo count can influence the outcome of downstream tasks [23]. As our focus is to identify the most influential species, the use of pseudo counts is justifiable. We show the results for Genus, Family and Order with a pseudo count of 0.5 to 1.0 with 0.01 increments to showcase the sensitivity following [24], c.f. Table S1 and Fig. S4.

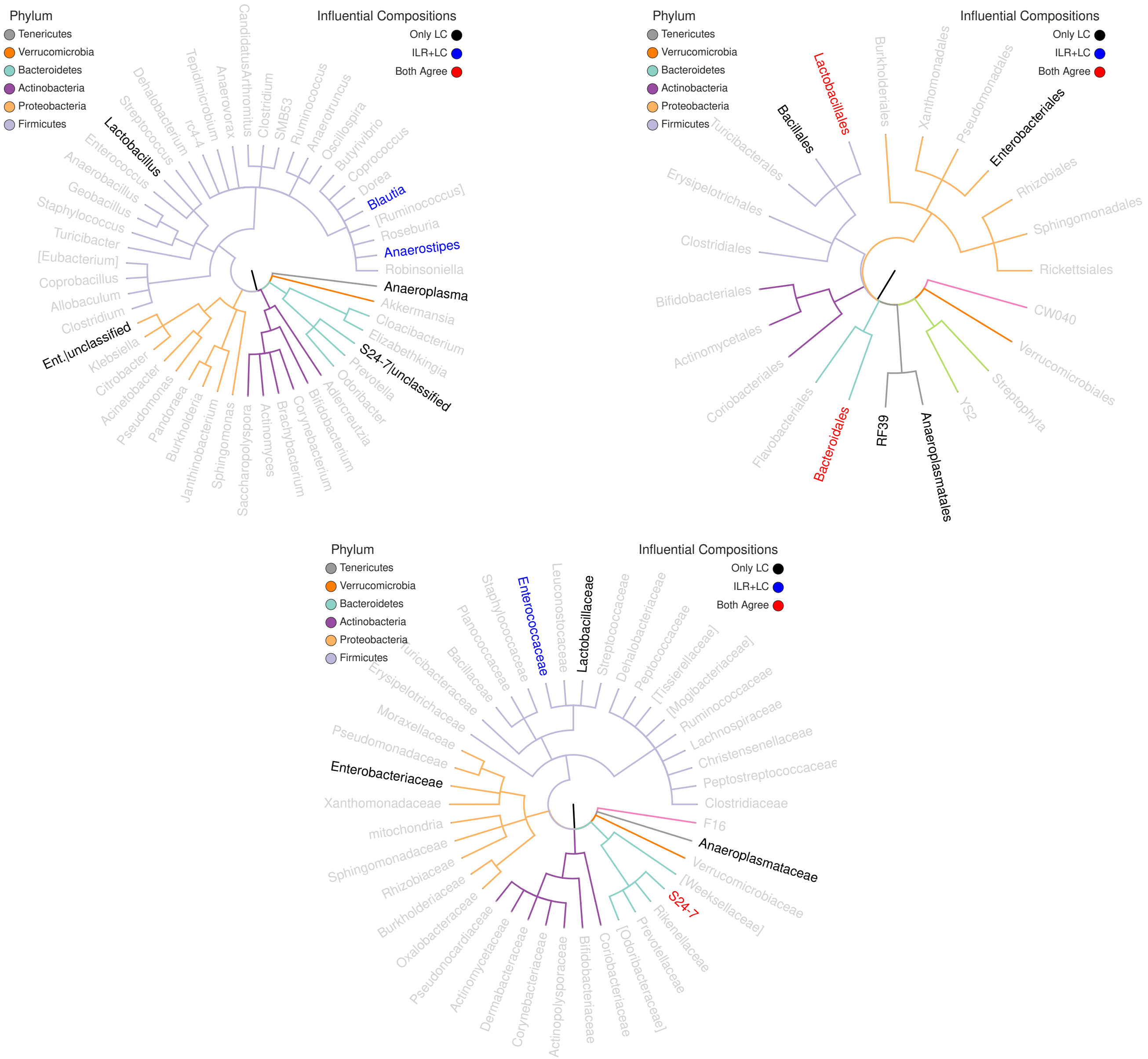

Figure S3. **Influential Compositions on Genus Level (top left), Order Level (top right) and Family Level (bottom):** In general, the results stay the same for ILR+LC in the binary case and the continuous case. The influential log-ratios are slightly shifting when simply applying a naive regression.

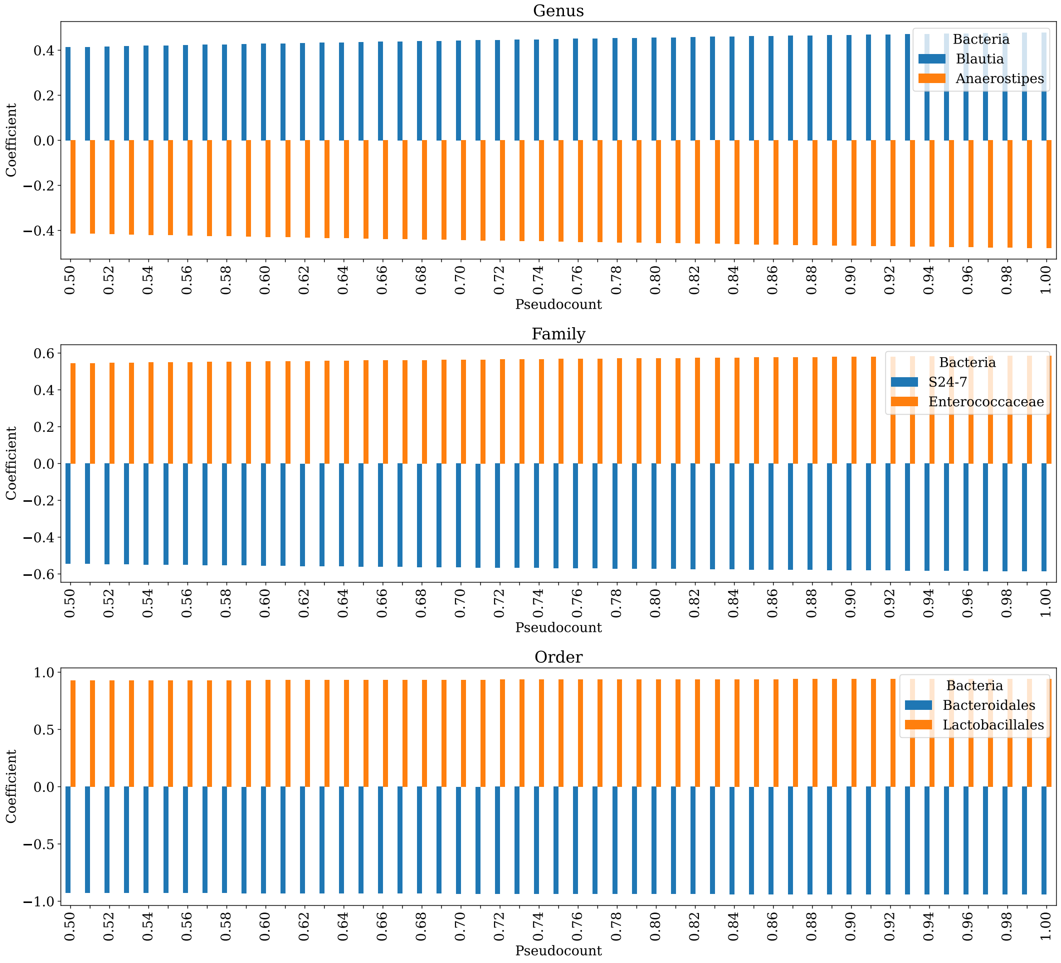

Figure S4. The barplots show the most influential log-ratio for different pseudo counts for different aggregation levels.

Genus	**Pseudo Count** 0.5	**Pseudo Count** 1.0
**Blautia**	0.413595	0.479329
**Anaerostipes**	-0.413595	- 0.479329

Order	**Pseudo Count** 0.5	**Pseudo Count** 1.0
**Lactobacillales**	0.926113	0.942751
**Bacteroidales**	-0.926113	- 0.942751

Family	**Pseudo Count** 0.5	**Pseudo Count** 1.0
**Enterococcaceae**	0.544683	0.586421
**S24-7**	-0.544683	-0.586421

Table S1. The results for the two most commonly used pseudo counts 0.5 and 1.0 show that while the influential composition does not change, the estimated influence does slightly increase with a higher pseudo count.

S6 Data Generation

This section describes the details of how we generate data for our empirical evaluation. Complementary to the real microbiome data, we consider several approaches to generate data for the compositional instrumental variable setting. Since counterfactuals are never observed in practice, we need a setup where the ground truth is known and can be controlled. We choose to simulate data from two different data generating models, Setting A and Setting B. The first one will put (most of) our models in a wellspecified setting, where we have strong expectations and theoretical guarantees on how they will behave. The other approach simulates compositional data by a zero-inflated negative binomial. Thus, the first stage of all of our models will be misspecified (except for potentially KIV assuming a proper choice of the kernels). This allows us to test our models for robustness and probe their limitations.

Based on this motivation, we also describe two additional parameter settings within Setting A that will examine robustness and limitations: a weak instrument scenario and a scenario with a non-linear second stage f. The first scenario will test the necessity of a strong/valid instrument, the second scenario will further look into the issue of misspecification (now in the second stage).

We describe the data generating model and the specific parameter settings. We also provide visualizations of the resulting data distributions, which is rather tricky for compositional data with $p > 3$. We will then supplement the result section of the main text with additional comments on the evaluation of the results and show the complete set of plots for Table 1 and Table 2.

Each generated dataset for $p = 3$ comprises $n = 1000$ samples, resp. 10,000 samples for $p = 30$ and $p = 250$, with an additional $n_{\text{intervention}} = 250$ interventional samples for evaluation of OOS MSE. Note that the examples in the figures show only one of these datasets. To ensure reproducibility, we consistently chose the 10th dataset of the confidence runs for (a representative) visualization.

Setting A

The following explanations refer to *Setting A* described in the main part.

Setting A generates data that enables us to assess our methods in a *wellspecified* setting. Instead of modeling $X \in \mathbb{S}^{p-1}$ directly, we model $\text{ilr}(X)$. The setting is strictly linear in $\text{ilr}(X)$. This means that both g and f are linear functions of U and Z, resp., U and $\text{ilr}(X)$. The generative model is as follows:

$$\begin{aligned} Z_j &\sim \text{Uniform}(0,1) \\ U &\sim \mathcal{N}(\mu_c, 1) \\ \text{ilr}(X) = g(Z,U) &= \alpha_0 + \alpha Z + c_X U \\ Y = f(X,U) &= \beta_0 + \beta^T \text{ilr}(X) + c_Y U \end{aligned} \tag{14}$$

Setting A with $p = 3, q = 2$

The main characteristics of this lower dimensional dataset are the presence of all microbes and relatively seldom zero values. We choose the following parameters for the low-dimensional case:

$$\mu_c = -3, \alpha_0 = [1,1], \alpha = \begin{bmatrix} 0.5 & -0.15 \\ 0.3 & 0.7 \end{bmatrix}, c_X = [0.5, 0.5], \beta_0 = 0.5, \beta = [4,1], c_Y = 4 \tag{15}$$

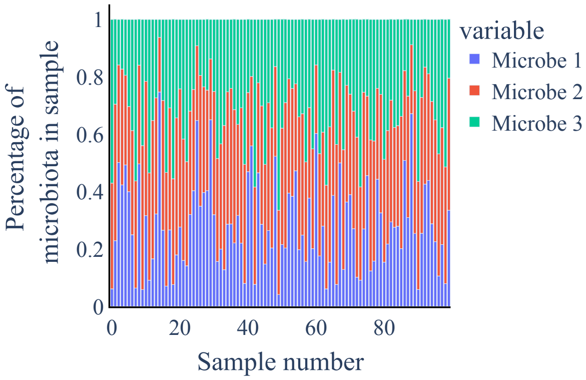

Figure S5. **Setting A with $p = 3, q = 2$:** The barplot shows the three-part composition of the first 100 samples. The microbes are evenly distributed over the individual compositions.

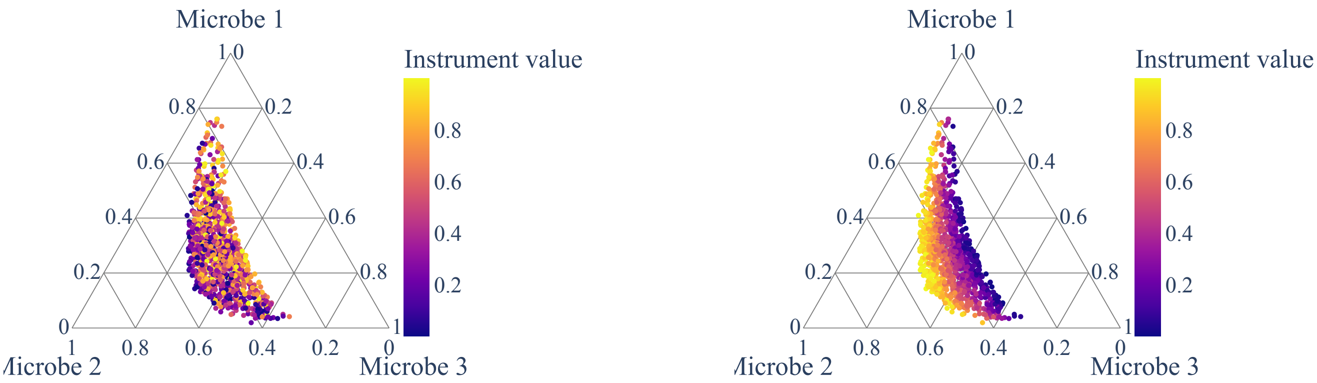

Figure S6. **Setting A with $p = 3, q = 2$:** The ternary plots are colored by first (left) and second (right) instrument value. The influence of Z_2 on the composition X is particularly pronounced and visually supports the assumption of Z being a valid instrument.

The first stage F-test for the two components of $\text{ilr}(X)$ gives $(32.18, 113.99)$ for the 10th data sample.

We remark that in higher dimensions, the F-test does not provide a strong theoretical justification for sufficient instrument strength, but we still use it as a sensible heuristic that provides a relative measure between different settings, i.e., in which scenario the instrument is stronger.

For the $p = 3$ case, we can visualize X by its compositional coordinates not only in a barplot (Figure S5) but also in an arguably more informative ternary plot (Figure S6). To visualize the linear relationship between observed $\text{ilr}(X)$ and Y as well as the true effect $Y \mid do(X)$, we transform the data X and visualize each component in a separate scatter plot (see Figure S7).

Setting A with $p = 30, q = 10$

Contrary to the previous example, we now analyze a slightly higher-dimensional setting with $p = 30$. In this scenario, it makes sense to introduce sparsity in the data generation process from a practical viewpoint. We work with the data generation setting given in Equation (14) and choose the following parameters:

$$\mu_c = 5,\ \alpha_0 = [3,1,1,1,3,1,1,1,0,\cdots,0],\ \alpha_{ij} \begin{cases} 0, & \text{for } i \neq j \text{ and } i,j > 8, \\ 1, & \text{for } i \neq j \leq 8 \end{cases},$$

$$c_X = [-2,-1,-1,-1,2,1,1,1,0,\cdots,0],\ \beta_0 = 5,\ \beta_{\log} = [10,5,5,5,-10,-5,-5,-5,0,\cdots,0],\ \beta = V^T \cdot \beta_{\log},\ c_Y = 5$$

for $i \in \{1,\ldots,p-1\}, j \in \{1,\ldots,q\}$ and V providing the orthonormal basis for the ilr-transformation (see Supplementary Material S2).

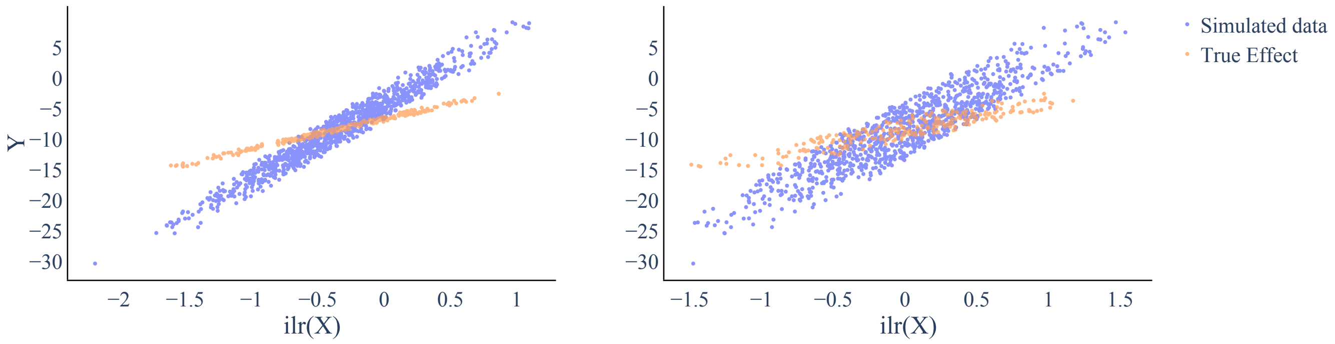

Figure S7. Setting A with $p = 3$, $q = 2$: Both plots show one component of $\text{ilr}(X) \in \mathbb{R}^2$ vs. the confounded outcome (blue) and the true effect (orange). Due to the confounding, the observed and the causal effect do not overlap. However, we expect the instrument Z to factor out the confounding effect and enable the two-stage methods to identify the causal effect.

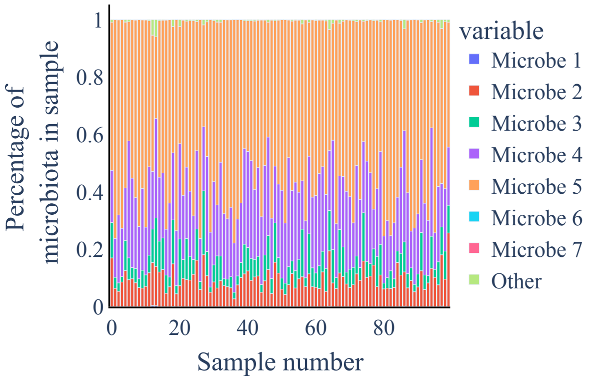

Figure S8. Setting A with $p = 30$, $q = 10$: The barplot shows the composition of the first 100 samples. The compositions are dominated by a few species.

Since a visualization with a ternary plot is no longer feasible, we only show barplots of the data in Figure S8. However, scatter plots showing individual $\text{ilr}(X)$ coordinates versus the observed Y and the true causal effect are still informative. Since the first components are the most influential ones in our setting, we show the first five $\text{ilr}(X)$ components in Figure S9.

Setting A with $p = 250, q = 10$

We now analyze the second high-dimensional setting with $p = 250$. As in the scenario of $p = 30$, it makes sense to introduce sparsity in the data generation process from a practical viewpoint. We work with the data generation setting given in Equation (14) and choose the following parameters:

$$\mu_c = 3,\ \alpha_0 = [1,1,3,1,1,1,3,1,1,1,3,1,0,\cdots,0],\ \alpha_{ij} \begin{cases} 0, & \text{for } i \neq j \text{ and } i,j > 8, \\ 1, & \text{for } i \neq j \leq 8 \end{cases},$$

$$c_X = [-1,2,-1,2,-1,2,-2,1,-2,1,-2,1,0,\cdots,0],\ \beta_0 = 5,\ \beta_{\log} = [10,5,5,5,-10,-5,-5,-5,0,\cdots,0],$$

$$\beta = V^T \cdot \beta_{\log},\ c_Y = 5$$

for $i \in \{1,\ldots,p-1\}, j \in \{1,\ldots,q\}$ and V providing the orthonormal basis for the ilr-transformation (see Supplementary Material S2). Since a visualization with a ternary plot is no longer feasible, we only show barplots of the data in Figure S10. However, scatter plots showing individual $\text{ilr}(X)$ coordinates versus the observed Y and the true causal effect are still informative. Since the first components are the most influential ones in our setting, we show the first five $\text{ilr}(X)$ components in Figure S11.

Setting B

The following explanations refer to *Setting B* described in the main part.

Setting B serves three main purposes: (i) to assess our methods on a dataset that closely resembles real-world data in terms of its distribution, (ii) to assess our methods when the first stage is misspecified, and (iii) to allow for sparsity in the first stage

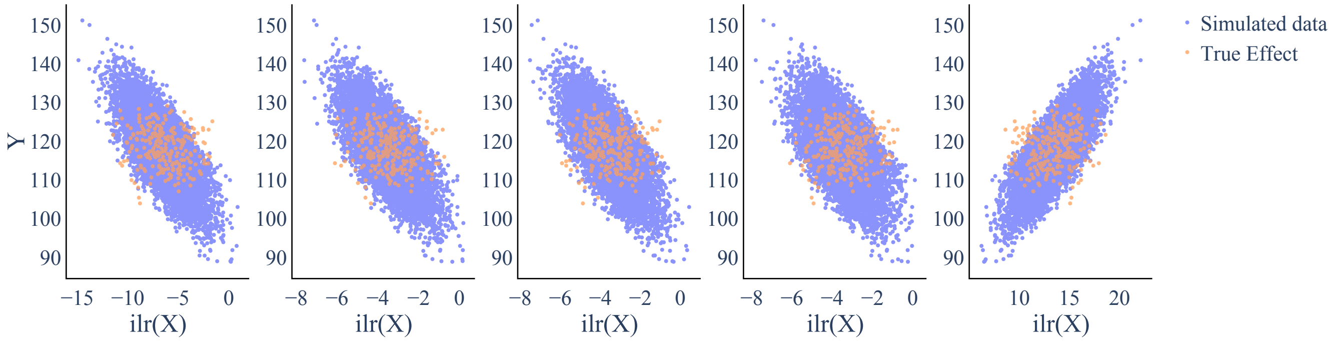

Figure S9. Setting A with $p = 30$**,** $q = 10$ **:** Both plots show one component of $\mathrm{ilr}(X) \in \mathbb{R}^{29}$ vs. the confounded outcome (blue) and the true effect (orange). Due to the confounding, the observed and the causal effect do not overlap. However, we expect the instrument Z to factor out the confounding effect and enable the two-stage methods to identify the causal effect.

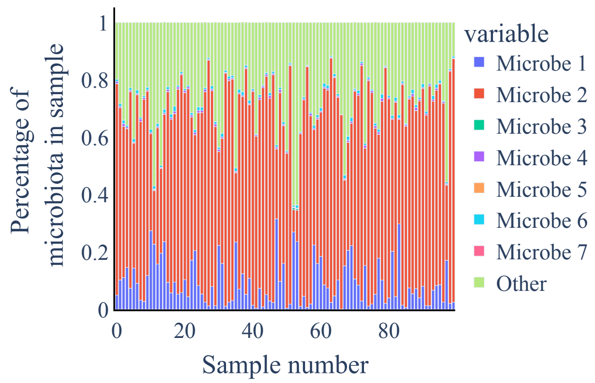

Figure S10. Setting A with $p = 250$**,** $q = 10$**:** The barplot shows the composition of the first 100 samples. The compositions are dominated by a few species.

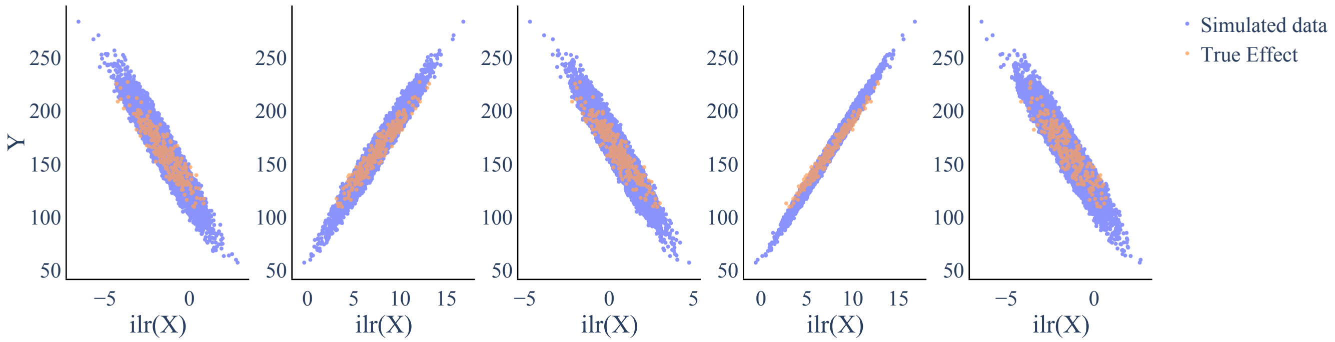

Figure S11. Setting A with $p = 250$**,** $q = 10$**:** Both plots show one component of $\mathrm{ilr}(X) \in \mathbb{R}^{249}$ vs. the confounded outcome (blue) and the true effect (orange). Due to the confounding, the observed and the causal effect do not overlap. However, we expect the instrument Z to factor out the confounding effect and enable the two-stage methods to identify the causal effect.

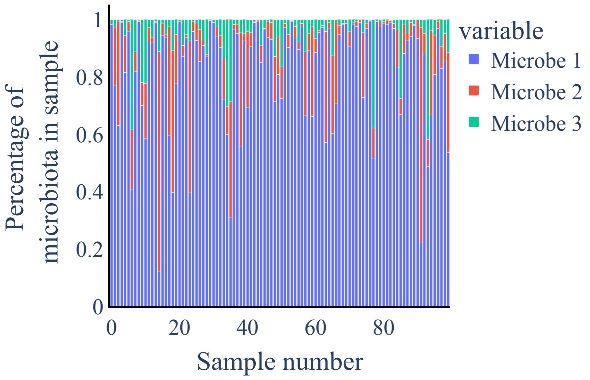

Figure S12. Setting B with $p = 3, q = 2$: The barplot shows the three-part composition of the first 100 samples. The data sample shows some dominating species in the individual compositions while having more variation between the samples compared to Setting A.

of the data generating process, resembling the real data in [1]. The sparsity of the compositional data can be accomplished by a zero-inflated negative binomial distribution. As ZINegBinomial is a frequently used distribution in modeling microbiome data, we assume a closer resemblance to real world sparsity than the resemblance we achieve in Setting A for $p = 30$ and $p = 250$.

The data is generated according to the following model with the parameter μ of the negative binomial as $\mu = \alpha_0 + \alpha Z$:

$$\begin{aligned} Z_j &\sim \text{Uniform}(Z_{\min}, Z_{\max}), \\ U &\sim \text{Uniform}(U_{\min}, U_{\max}), \\ X = g(Z,U) &\sim C(\text{ZINegBinomial}(\mu, \Sigma, \theta, \eta)) \oplus (\Omega_C \odot U), \\ Y = f(X,U) &= \beta_0 + \beta^T \log(X) + c_Y^T \log(\Omega_C \odot U) \end{aligned} \tag{16}$$

We fix $Z_{\min} = 1, Z_{\max} = 10$ and $U_{\min} = 0.2, U_{\max} = 3$ throughout. For the negative binomial distribution we set $\Sigma = \mathbb{I}_p$, i.e., assuming no additional correlation within the different components of the composition for simplicity.

Setting B with $p = 3, q = 2$

The parameter setting with $p = 3$ does not yet contain sparse data due to its low-dimensionality. It serves the purpose to compare the performance of the two-stage methods in a misspecified setting and a wellspecified setting (except for DIR+LC which is misspecified in both Setting A and Setting B).

Here, we consider the following generative model based on Equation (16). We fix $Z_{\min} = 0, Z_{\max} = 10, U_{\min} = 0.2, U_{\max} = 3$. We chose α_0 to be $[7, 9, 8]$ and $\alpha = \begin{bmatrix} 5 & 0 & 0 \\ 0 & 5 & 0 \\ 0 & 0 & 5 \end{bmatrix}$ to guarantee for valid instruments. We set the dispersion to $\theta = 2$ and keep the zero probability at $\eta = [0, 0, 0]$ to get valid compositions for this low-dimensional scenario. For the confounding composition Ω_C, we set it to $[0.7, 0.1, 0.2]$. For the second stage, we fix ground truth parameters $\beta_0 = 1$, $\beta_{\log} = [-5, 3, 2]$, which results in $\beta = V^T \beta_{\log}$ and the confounding parameter $c_Y = [2, -10, -10]$.

The first stage F-test for the two components of $\text{ilr}(X)$ gives $(41.38, 14.08)$ for the 10th data sample. We remark that in higher dimensions, the F-test does not provide a strong theoretical justification for sufficient instrument strength, but we still use it as a sensible heuristic that provides a relative measure between different settings, i.e., in which scenario the instrument is stronger.

For the $p = 3$ case, we can visualize X by its compositional coordinates not only in a barplot (Figure S12) but also in an arguably more informative ternary plot (Figure S13). To visualize the relationship between observed $\text{ilr}(X)$ and Y as well as the true effect $Y \mid do(X)$, we transform the data X and visualize each component in a separate scatter plot (see Figure S14).

Setting B with $p = 30, q = 10$

In the higher-dimensional scenarios we will make us of the sparsity ability of the ZINegBinom distribution.

The parameters were chosen to generate a suitable dataset that still conveys typical compositional data properties (sparsity, high variance within the composition, similar means to real data) and significant instruments. Here, we consider the following generative model based on Equation (16). We fix $Z_{\min} = 0, Z_{\max} = 10, U_{\min} = 0.2, U_{\max} = 3$. To ensure a handful of components

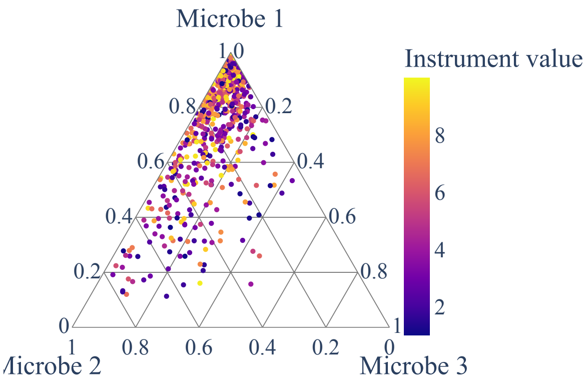

Figure S13. Setting B with $p = 3$, $q = 2$: The ternary plots are colored by first (left) and second (right) instrument value. Due to the data generation process, the influence of Z_1 and Z_2 on the composition X is less visually obvious than for Setting A. Nevertheless, Z can be assumed to be a valid instrument.

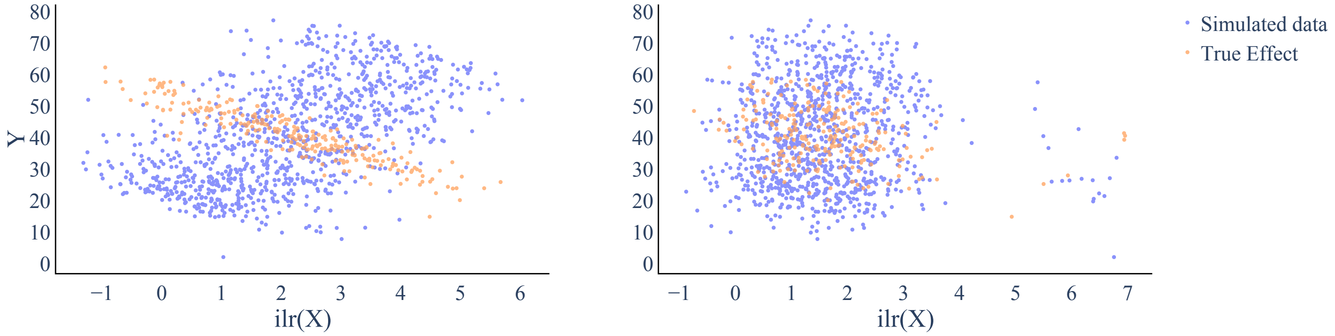

Figure S14. Setting B with $p = 3$, $q = 2$: Both plots show one component of $\mathrm{ilr}(X) \in \mathbb{R}^2$ vs. the confounded outcome (blue) and the true effect (orange). Due to the confounding, the observed and the causal effect do not overlap. However, we expect the instrument Z to factor out the confounding effect and enable the two-stage methods to identify the causal effect.

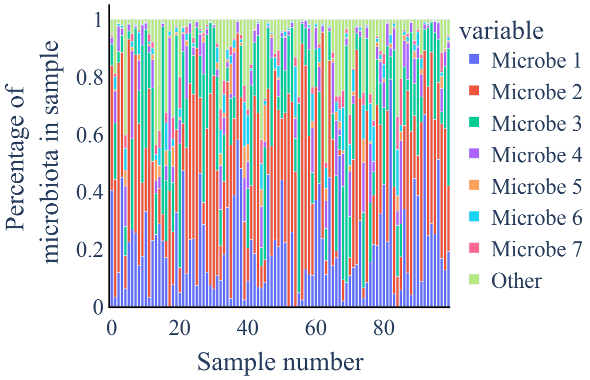

Figure S15. Setting B with $p = 30$**,** $q = 10$**:** The barplot shows the different compositions of the first 100 samples in the dataset. We observe some dominating components and many small components with an overall high variability.

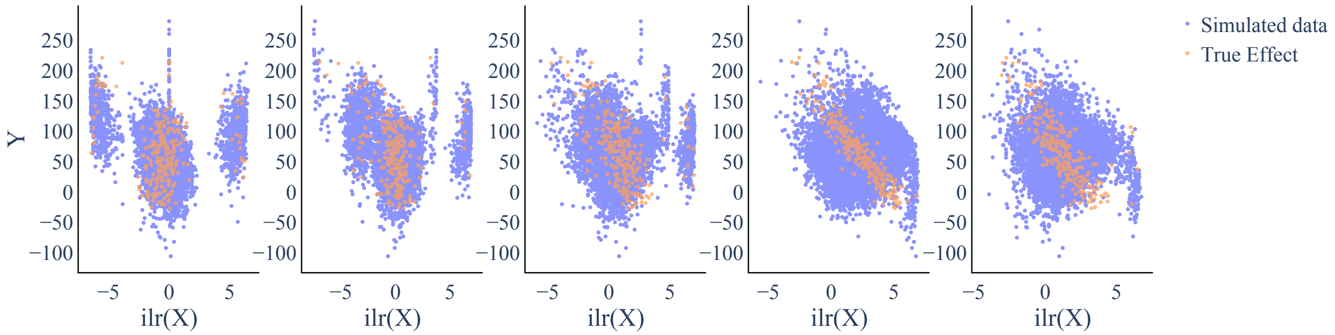

Figure S16. Setting B with $p = 30$**,** $q = 10$**:** Each plot shows one of the first five components of $\text{ilr}(X) \in \mathbb{R}^{29}$ vs. the confounded outcome (blue) and the true effect (orange). The dataset shows strong confounding in some of the components as the true effect and the observed effect actually contradict each other. We expect the two-stage methods to perform better than the naive regression in such scenarios. We can thus check if the two-stage methods are still able to make use of the instrument Z despite the misspecified first stage.

dominating the composition, we fix the first 8 entries of α_0 to be $[1,1,2,1,4,4,2,1,4,4,2,1]$ and randomly sample the remaining ones from UniformChoice$([1,2,2])$. For α, which mainly controls the instrument strength, we use a deterministic value to guarantee valid instruments:

$$\alpha_{ij} \begin{cases} 0, & \text{for } i \neq j \text{ and } i, j > 8, \\ 1, & \text{for } i \neq j \leq 8 \end{cases} \tag{17}$$

We set the dispersion to $\theta = 2$ and the zero probability value $\eta = [0, \ldots, 0, 0.8, \ldots, 0.8]$. For the confounding composition Ω_C, we fix the first components to $[0.2, 0.3, 0.2, 0.1]$, to ensure that the most dominating parts of the composition are also more strongly influenced by confounding. Then we sample the remaining components of Ω_C from UniformChoice$([0.01, 0.05])$ and eventually apply the closure operator C to ensure Ω_C is a composition. For the second stage, we fix ground truth parameters $\beta_0 = 1$, $\beta_{\log} = [-10, -5, -5, -5, 10, 5, 5, 5, 0, \ldots, 0]$, which results in $\beta = V^T \beta_{\log}$ and the confounding parameter $c_Y = [10, 10, 5, 15, -5, -5, -5, -5, -5, -5, -5, -5, 0, \ldots, 0]$.

For a brief overview, we visualize the first five components of the $\text{ilr}(X)$ coordinates versus the observed Y and the true causal effect in Figure S16 and show barplots of the generated data in Figure S15.

Setting B with $p = 250, q = 10$

We consider now the second high-dimensional scenario for Setting B with $p = 250$. The parameters for Setting B with $p = 250$ are very close to the parameters for Setting B with $p = 30$.

Again, we consider the following generative model based on Equation (16). We fix $Z_{\min} = 0, Z_{\max} = 10, U_{\min} = 0.2, U_{\max} = 3$. To ensure a handful of components dominating the composition, we fix the first 8 entries of α_0 to be $[1,1,2,1,4,4,2,1,4,4,2,1]$

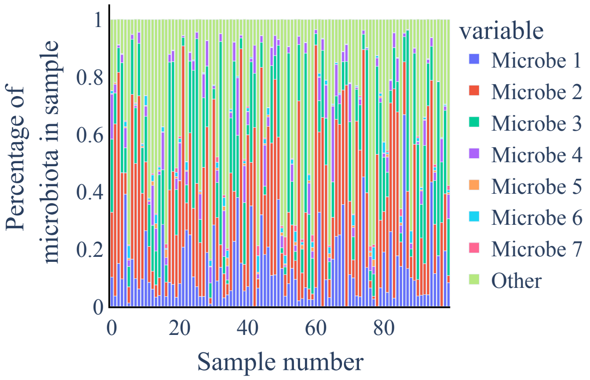

Figure S17. Setting B with $p = 250$, $q = 10$: The barplot shows the different compositions of the first 100 samples in the dataset. We still observe a few dominating components and many small components with an overall high variability.

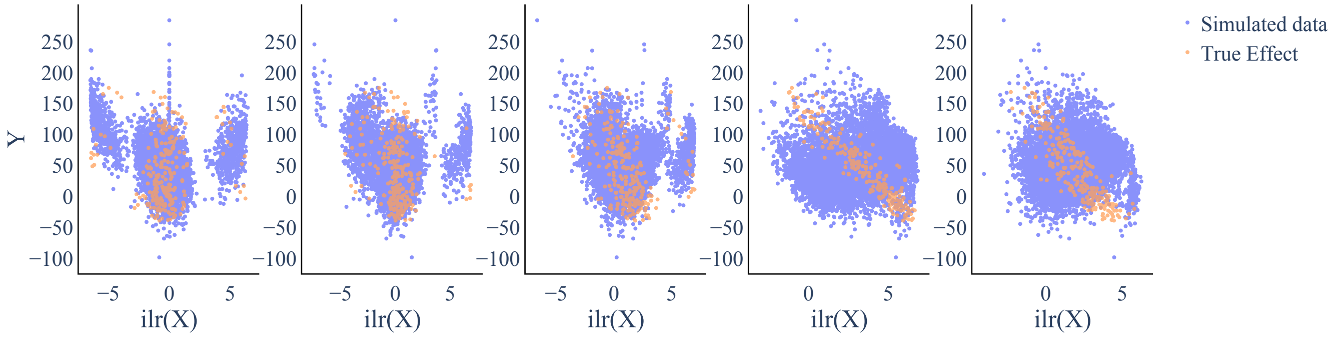

Figure S18. Setting B with $p = 250$, $q = 10$: Each plot shows one of the first five components of $\mathrm{ilr}(X) \in \mathbb{R}^{249}$ vs. the confounded outcome (blue) and the true effect (orange). The dataset shows strong confounding in some of the components as the true effect and the observed effect actually contradict each other. We expect the two-stage methods to perform better than the naive regression in such scenarios. We can thus check if the two-stage methods are still able to make use of the instrument Z despite the misspecified first stage.

and randomly sample the remaining ones from $\mathrm{UniformChoice}([1,2,2])$. For α, which mainly controls the instrument strength, we use a deterministic value to guarantee valid instruments:

$$\alpha_{ij} \begin{cases} 0, & \text{for } i \neq j \text{ and } i,j > 8, \\ 1, & \text{for } i \neq j \leq 8 \end{cases} \tag{18}$$

We set the dispersion to $\theta = 2$ and the zero probability value $\eta = [0,\ldots,0,0.8,\ldots,0.8]$. For the confounding composition Ω_C, we fix the first components to $[0.2,0.3,0.2,0.1]$, to ensure that the most dominating parts of the composition are also more strongly influenced by confounding. Then we sample the remaining components of Ω_C from $\mathrm{UniformChoice}([0.01,0.05])$ and eventually apply the closure operator C to ensure Ω_C is a composition. For the second stage, we fix ground truth parameters $\beta_0 = 1$, $\beta_{\log} = [-10,-5,-5,-5,10,5,5,5,0,\ldots,0]$, which results in $\beta = V^T\beta_{\log}$ and the confounding parameter $c_Y = [10,10,5,15,-5,-5,-5,-5,-5,-5,-5,-5,0,\ldots,0]$.

For a brief overview, we visualize the first five components of the $\mathrm{ilr}(X)$ coordinates versus the observed Y and the true causal effect in Figure S18 and show barplots of the generated data in Figure S17.

Further Settings for Robustness Evaluation

By assuming a misspecified first stage in Setting B via the ZINegBinom distribution, we already started to evaluate the robustness of our methods. Nevertheless, we will further relax different requirements within Setting A. We evaluate the robustness via two additional scenarios

1. We relax the assumption of a valid instrument and test the sensitivity of the methods with respect to weak instruments.

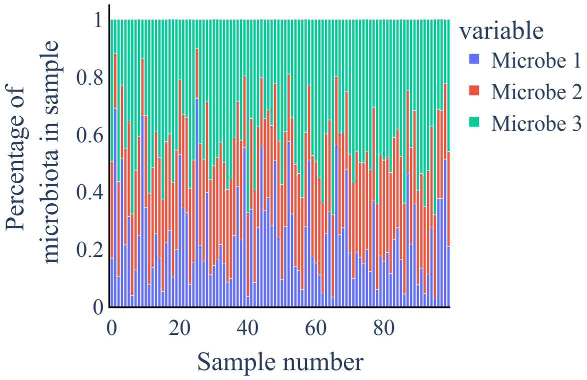

Figure S19. Setting A with $q = 2$, $p = 3$ and weak instruments: The barplot shows the different composition in each sample (plotted here for the first 100 samples). Microbe 2 has a relatively small value whereas microbe 1 and microbe 3 dominate the composition by high variation.

2. We assume a non-linear ground truth relationship f for the second stage, a scenario for which all the considered models are misspecified.

Weak Instrument

"Strong instruments" resp. "valid instruments" are a prerequisite for successful two-stage estimation and one of the key discussion points in applications of two-stage instrumental variable estimation. Instrument strength for $p = 1$ is typically measured via the first-stage F-statistic with a value > 10 being considered sufficient to avoid weak instrument bias in 2SLS [25]. For $p > 1$, measuring instrument strength is not as straightforward [26] and we thus report F-statistics for each dimension of the treatment (either $X \in \mathbb{S}^{p-1}$ or $\mathrm{ilr}(X) \in \mathbb{R}^{p-1}$) separately. Theoretically, the estimation bias can become arbitrarily large (even in the large data limit) for weak instruments. To quantitatively assess the effect of weak instruments in our specific applications, we provide an additional simulation scenario and its results (see Supplementary Material S8) for a weak instrument settings.

Setting A with $p = 3, q = 2$ and weak instruments For testing in a weak instrument setting, we return to Setting A. We mostly control the instrument strength via α and use higher or lower α values to obtain a strong or weak instrument setting. We choose the following parameters for a weak instrument:

$$\mu_c = -2, \alpha_0 = [4,1], \alpha = \begin{bmatrix} 0.05 & 0.01 \\ 0.2 & 0 \end{bmatrix}, c_X = [1,1], \beta_0 = 2, \beta = [6,2], c_Y = 4 \tag{19}$$

The first stage F-test for the two components of $\mathrm{ilr}(X)$ gives $(6.9, 4.7)$, much weaker than the previous settings. Again we show a barplot (Figure S19) and a ternary plot (Figure S20) of the generated data. The observed data as well as the true causal effect are shown in Figure S21.

Nonlinear Second Stage

Contrary to the previous scenarios, we now consider a non-linear f, resulting in a misspecified second stage for most of our methods. Note that in this scenario all two-stage methods as well as the naive regression will be misspecified in the second stage.

Setting A with $p = 3, q = 2$ and non-linear f Specifically, we replace the linear function for Y in Equation (14) with

$$Y = \beta_0 + \frac{1}{100} \mathbf{1}^T (\mathrm{ilr}(X) + 1)^3 + 10 \cdot \mathbf{1}^T \sin(\mathrm{ilr}(X)) + c_Y U. \tag{20}$$

The remaining parameters are chosen to yield a strong instrument, ensuring that any performance differences are not (in addition) due to weak instrument bias:

$$\mu_c = -1, \alpha_0 = [1,1], \alpha = \begin{bmatrix} 4 & 1 \\ -1 & 3 \end{bmatrix}, c_X = [2,2]\beta_0 = 5, \beta = [6,2], c_Y = 4 \tag{21}$$

Note that in this setting β cannot be interpreted directly as the causal parameters, since the true causal effect also has a non-linear dependence on $\mathrm{ilr}(X)$. Since the first stage remains unchanged, we can still use an F-test to assess instrument

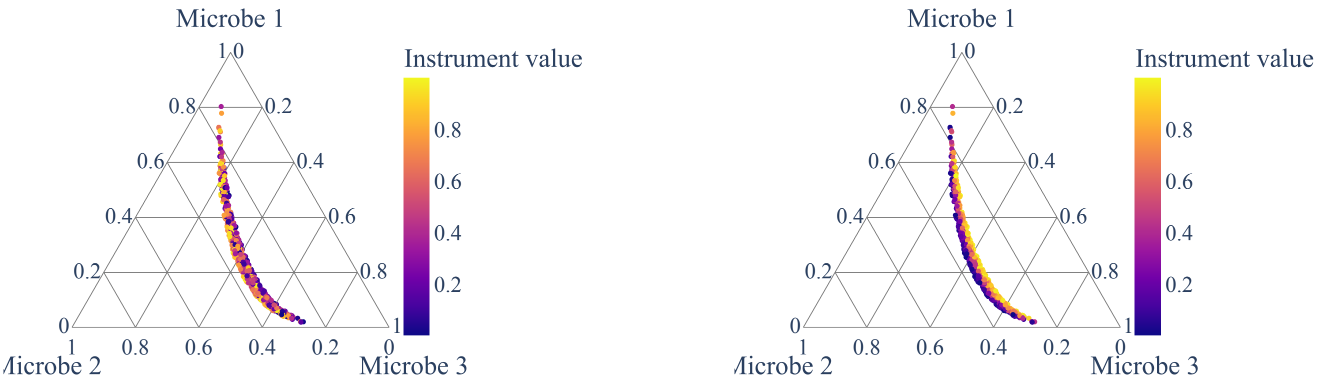

Figure S20. Setting A with $q = 2$, $p = 3$ and weak instruments: The ternary plots are colored by first (left) and second (right) instrument. The composition of the instrument is barely influenced by the value of Z.

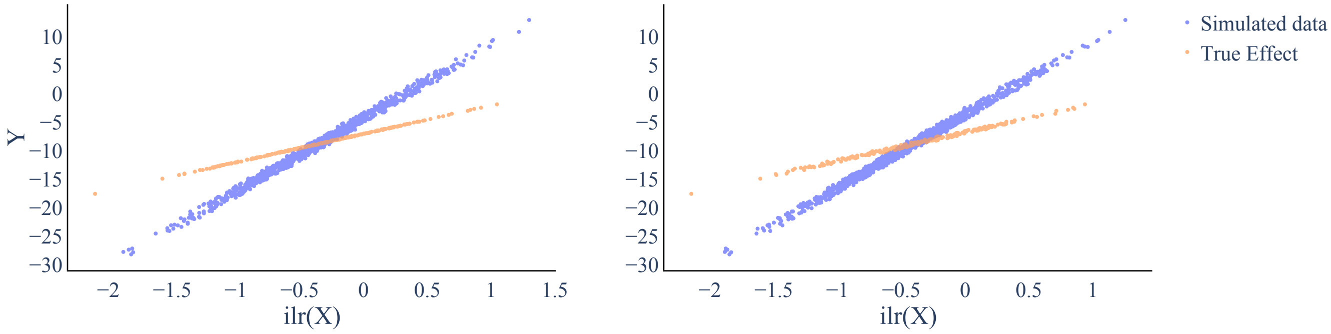

Figure S21. Setting A with $q = 2$, $p = 3$ and weak instruments: Both plots show one component of $\text{ilr}(X) \in \mathbb{R}^2$ vs. the confounded outcome (blue) and the true effect (orange). Due to the confounding, both effects do not overlap. As we are in the weaker instrument setting, we expect the methods to perform not as stable as in the previous cases where we had a stronger instrument available.

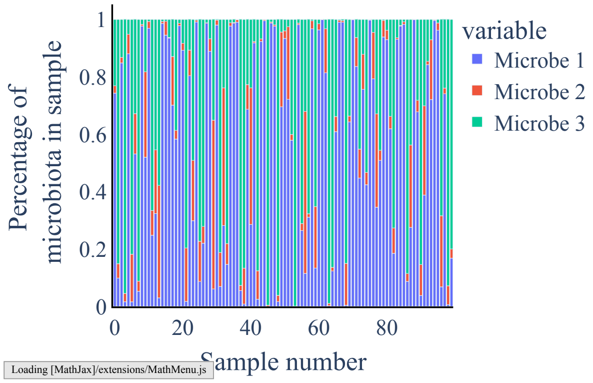

Figure S22. Setting A with $p = 3, q = 2$ and a non-linear function form of f: The barplot shows the different composition for each sample (for the 100 first data points). Microbe 1 and 2 dominate the composition with high variance.

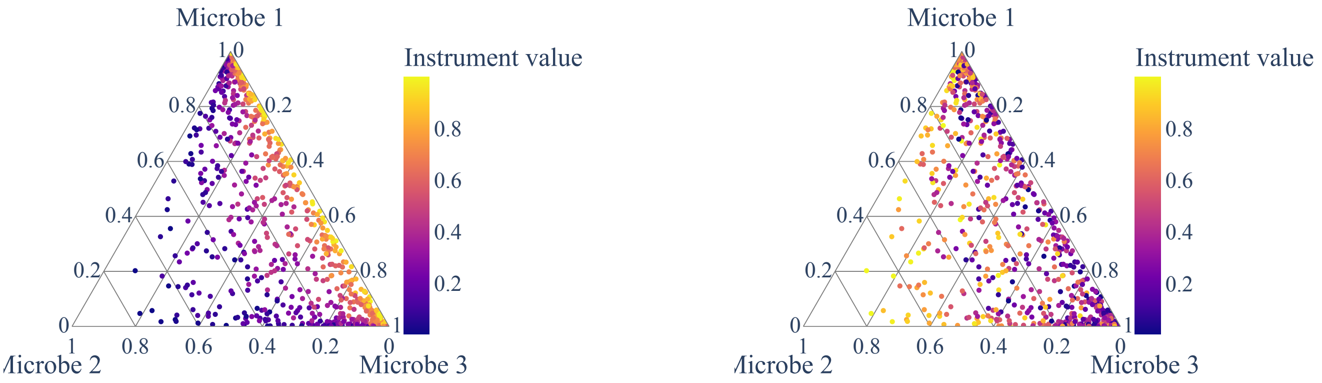

Figure S23. Setting A with $p = 3, q = 2$ and a non-linear function form of f: The ternary plots for the non-linear setup with $q = 2$, colored by first (left) and second (right) instrument. Note that the first stage is still linear in $\mathrm{ilr}(X)$. Thus, the generation of the X values is not affected by the change in f.

strength, which results in $(164.7, 76.4)$, a solid indicator for a strong instrument. Again we show a barplot (Figure S22) and a ternary plot (Figure S23) of the generated data. The observed data as well as the true causal effect are shown in Figure S24.

Scarce Data Example $p \gg n$

We return to Setting A with linear dependencies in both stages. However, in the scenarios before, we assumed a large dataset ($n = 10{,}000$) for the methods to work on. In many real applications, this might not be the case. Thus we choose to include an additional robustness aspect concentrating on the scenario $p \gg n$. In this particular case we chose $p = 250$ and $n = 100$.

Setting A with $p = 250, q = 10$ **and** $n = 100$ The choice of parameter is the same to Setting A with $n = 10{,}000$, however we only include the first 100 samples for the estimation:

$$\mu_c = 3,\ \alpha_0 = [1,1,3,1,1,1,3,1,1,1,3,1,0,\cdots,0],\ \alpha_{ij} \begin{cases} 0, & \text{for } i \neq j \text{ and } i,j > 8, \\ 1, & \text{for } i \neq j \leq 8 \end{cases},$$

$$c_X = [-1,2,-1,2,-1,2,-2,1,-2,1,-2,1,0,\cdots,0],\ \beta_0 = 5,\ \beta_{\log} = [10,5,5,5,-10,-5,-5,-5,0,\cdots,0],$$
$$\beta = V^T \cdot \beta_{\log},\ c_Y = 5$$

for $i \in \{1,\ldots,p-1\}, j \in \{1,\ldots,q\}$ and V providing the orthonormal basis for the ilr-transformation (see Supplementary Material S2).

For the sake of completeness, we show a barplot (Figure S25) of the generated data. We note that the samples are the first 100 samples of the larger dataset of the original Setting A with $p = 250, q = 10$ and $n = 10{,}000$. The observed data as well as

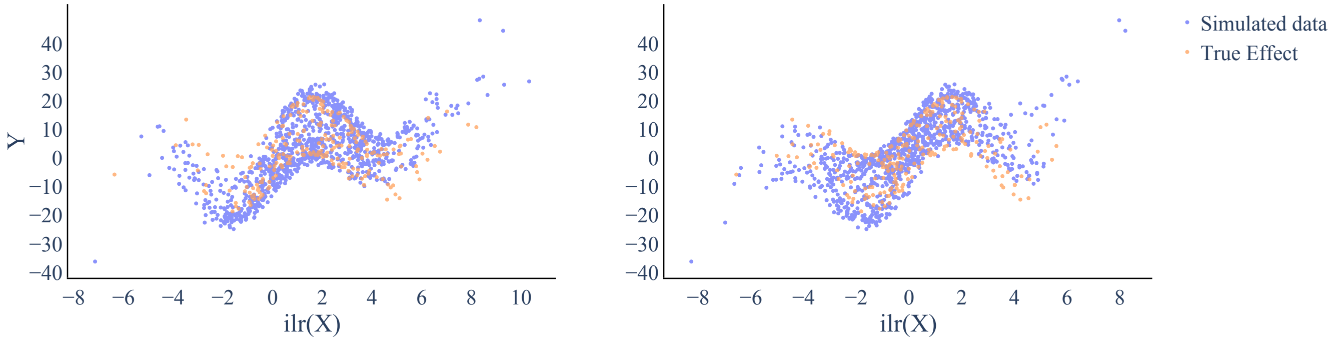

Figure S24. Setting A with $p = 3$, $q = 2$ and a non-linear function form of f: Both plots show one component of $\mathrm{ilr}(X) \in \mathbb{R}^2$ vs. the confounded outcome (blue) and the true effect (orange). The effect both of the confounded outcome and the true effect show a non-linear dependency towards the individual $\mathrm{ilr}(X)$ components.

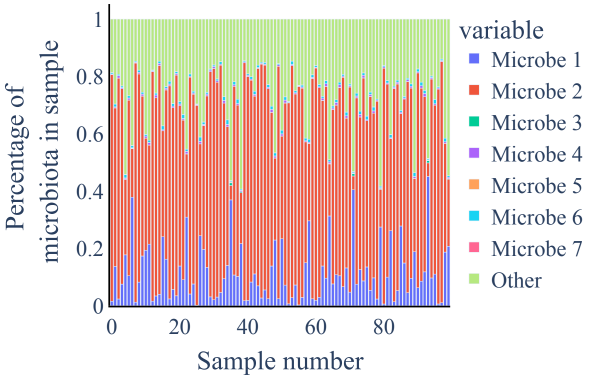

Figure S25. Setting A with $n = 100$, $p = 250$, $q = 10$: The barplot shows the composition of the 100 samples. The compositions are dominated by a few species.

the true causal effect are shown in Figure S26.

S7 Method Training

Dirichlet Regression

The mean of the Dirichlet distribution is given by $\mu_{\text{Diri}} = \frac{\alpha_j}{\sum_{j=1}^p \alpha_j}$. Here, we consider the following model for the mean components

$$\mathbb{E}[X_{ij}] = \frac{\alpha_j}{\sum_{j=1}^p \alpha_j} = \frac{\alpha_j(Z_i)}{\sum_{j=1}^p \alpha_j(Z_i)} \tag{22}$$

$$\log(\alpha_j(Z_i)) = \omega_{0j} + \omega_j Z_j. \tag{23}$$

The maximum likelihood function is then given by

$$l(\alpha; X, Z) = \frac{1}{n}\sum_{i=1}^n \log\Gamma\Big(\sum_{j=1}^p \exp\{\omega_{0j} + \omega_j Z_i\}\Big) \tag{24}$$

$$+ \frac{1}{n}\sum_{i=1}^n\sum_{j=1}^p \left(\log(X_{ij})\Big(\exp\{\omega_{0j} + \omega_j Z_i\} - 1\Big) - \log\Gamma\Big(\exp\{\omega_{0j} + \omega_j Z_i\}\Big)\right). \tag{25}$$

Additionally, we introduce a sparsity enforcing regularization term to arrive at the following objective function

$$\min_{\omega} -l(\alpha; X, Z) + \lambda_{\text{dirichlet}} \sum_{j=1}^p |\omega_j| \tag{26}$$

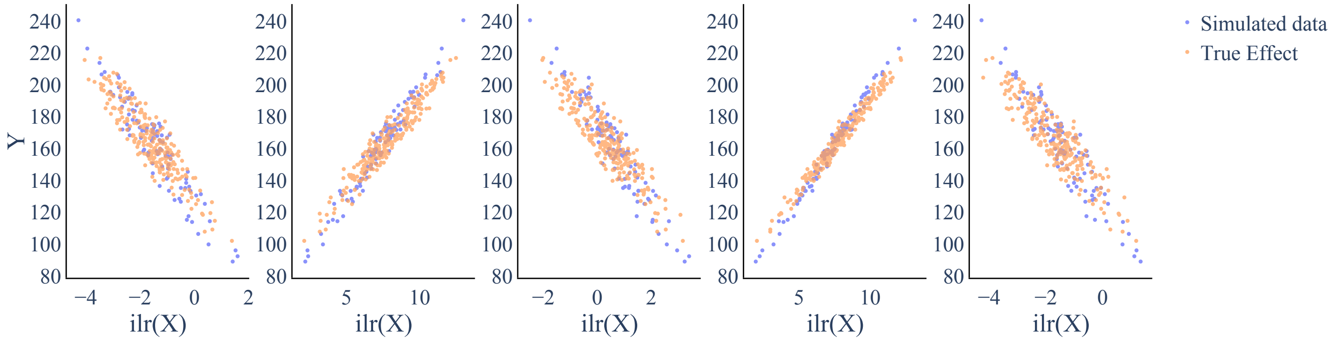

Figure S26. Setting A with $n = 100$, $p = 250$, $q = 10$: Both plots show one component of $\mathrm{ilr}(X) \in \mathbb{R}^{249}$ vs. the confounded outcome (blue) and the true effect (orange). Due to the confounding, the observed and the causal effect do not overlap. However, we expect the instrument Z to factor out the confounding effect and enable the two-stage methods to identify the causal effect. Note that now we consider a sample size of $n = 100$.

with $\lambda_{\text{dirichlet}} \geq 0$. For each Dirichlet regression, we pick $\lambda_{\text{dirichlet}}$ from the set $\{0.1, 1, 2, 5, 10\}$ by model selection via the Bayesian Information Criterion ($BIC = q \cdot \log(n) - 2 \cdot (\hat{L})$, with $\hat{L}$ being the likelihood value). We train the model for each available λ value in the set and choose the model with minimal BIC. For the starting point α_{start} we fit a Dirichlet distribution on those X for which all $|Z| < 0.2$ by maximum likelihood estimation.

Log-contrast Regression

The log-contrast regression is enforcing sparsity via an ℓ_1 penalty on the β parameters.

$$\min_{\beta} \sum_{i=1}^{n} \mathscr{L}(x_i, y_i, \beta) + \lambda \|\beta\|_1 \quad \text{s.t.} \sum_{i=1}^{p} \beta_i = 0\,. \tag{27}$$

This estimation respects the compositional nature of x while retaining the association between the entry β_i and the relative abundance of the individual component x_i.

In our examples, we focus mainly on continuous $y \in \mathbb{R}$ and the squared loss $\mathscr{L}(x, y, \beta) = (y - \beta^T \log(x))^2$. However, the framework also supports different loss functions.

For robust Lasso regression, the Huber loss can be applied.

$$\mathscr{L}(x_i, y_i, \beta) = \mathscr{H}_\delta(x_i, y_i, \beta) = \begin{cases} \frac{1}{2}(y_i - \beta^T \log(x_i))^2 & \text{for } |y_i - \beta^T \log(x_i)| < \delta \\ \delta(|y_i - \beta^T \log(x_i)| - \frac{1}{2}\delta), & \text{otherwise.} \end{cases} \tag{28}$$

The Huber Loss combines the squared loss and the absolute loss. It is less sensitive to outliers than the squared loss, but remains differentiable at 0 in contrast to the absolute loss.

Moreover, for classification tasks with $y_i \in \{-1, 1\}$, we can directly use the squared Hinge loss for $\mathscr{L}$ with:

$$\mathscr{L}(x_i, y_i, \beta) = l(x_i, y_i, \beta) \text{ with } l(x_i, y_i, \beta) = \begin{cases} (1 - (y_i \beta^T \log(x_i))^2, & \text{if } y_i \beta^T \log(x_i) \leq 1 \\ 0, & \text{if } y_i \beta^T \log(x_i) > 1 \end{cases} \tag{29}$$

or a "Huberized" version thereof:

$$\mathscr{L}(x_i, y_i, \beta) = l_\delta(x_i, y_i, \beta) \text{ with } l_\delta(x_i, y_i, \beta) = \begin{cases} (1 - (y_i \beta^T \log(x_i))^2, & \text{if } \delta \leq y_i \beta^T \log(x_i) \leq 1 \\ (1 - \delta)(1 + \delta - 2 y_i \beta^T \log(x_i)), & \text{if } y_i \beta^T \log(x_i) \leq \delta \\ 0, & \text{if } y_i \beta^T \log(x_i) > 1 \end{cases} \tag{30}$$

We refer to [17] for further loss functions and a more detailed overview.

We now continue with the description of the setup used in the following result section. The results on the synthetic data and the real data in Supplementary Material S8 are based on the squared loss:

$$\min_{\beta} \sum_{i=1}^{n} \|y_i - \beta^T \log(x_i)\|_2^2 + \lambda \|\beta\|_1 \quad \text{subject to} \sum_{i=1}^{p} \beta_i = 0\,. \tag{31}$$

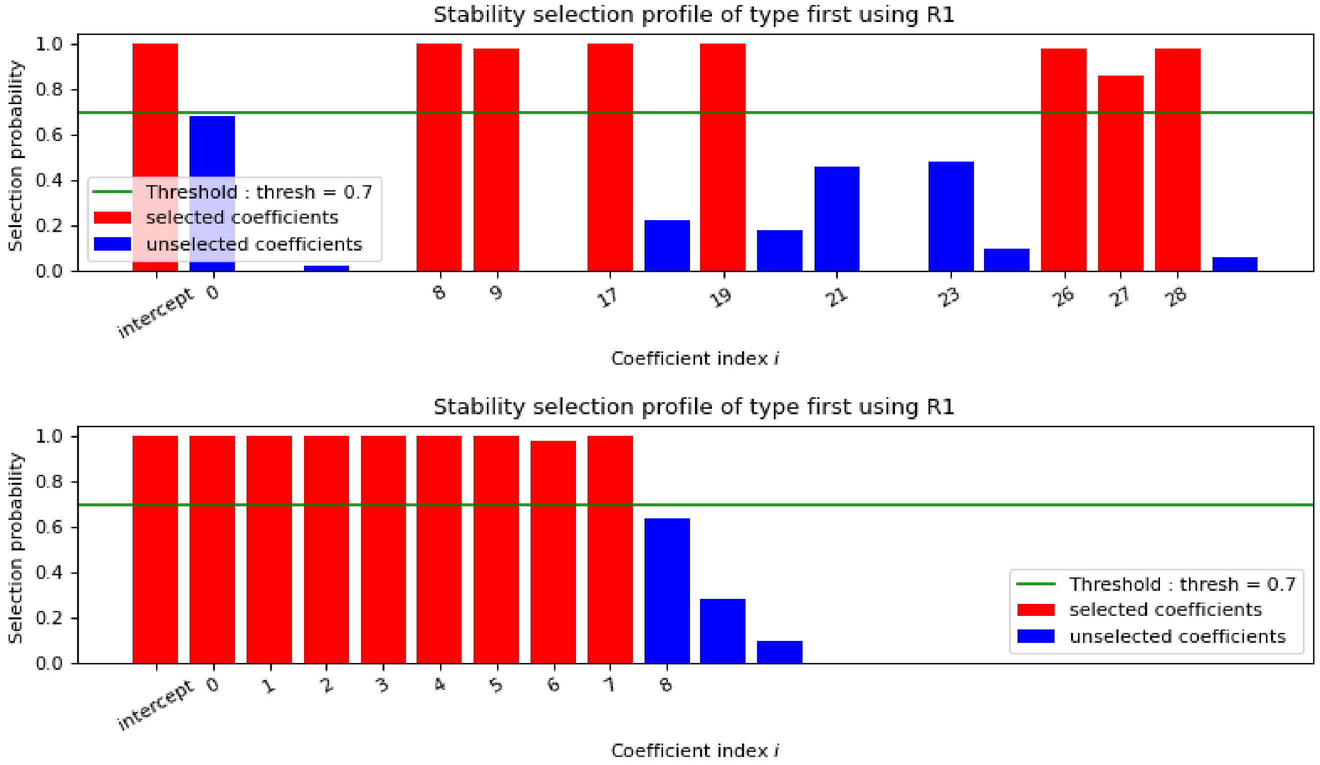

Figure S27. Stability profiles for sparse log contrast regression with c-lasso: The barplots show the model selection probability of the β coefficients. The upper panel shows the example for the naive regression. The lower panel shows the results for the same setting for ILR+LC regression. Both models are fairly certain about the main drivers.

Furthermore, for the real data we also show the results for a binary outcome $y_i \in \{-1, 1\}$ based on the squared Hinge loss (Equation (29)).

We solve the underlying optimization problems with the c-lasso package, a Python package for constrained sparse regression [17]. The c-lasso packages comprises several model selection schemes, including a theoretically-derived λ_0 parameter, k-fold cross-validation, and stability selection.

Here, we consider stability selection for tuning λ. The method comprises the hyperparameter $t_{\text{threshold}}$ which determines the number of coefficients included in the final model. In our training, we set the same $t_{\text{threshold}}$ for the naive regression as well as the two-stage methods to have a fair comparison. In all our training scenarios with generated data we find $t_{\text{threshold}} = 0.7$ to be a reasonable default value. For the real data scenario we found $t_{\text{threshold}} = 0.65$ to be more sensible.

We use Setting B with $p = 30$ and $q = 10$ as a representative example to illustrate the impact of the threshold value. Figure S27 shows the stability profile of the β coefficients and their attributed probability of entering the model. The threshold value $t_{\text{threshold}} = 0.7$ works as a cut off for the relevant coefficients. The upper panel shows the results for the naive regression, whereas the lower panel shows the results for the ILR+LC regression (working on the exact same data).

Moreover, the method also returns the coefficient values across the λ-path, i.e., the entry of coefficients into the model for the corresponding λ (see Figure S28). Further improvements may be achieved by taking the path and individual analysis into account instead of proposing a general $t_{\text{threshold}}$, however, this simple yet effective approach was sufficient for our purposes in this work.

S8 Method Results

For the comparison of the different methods, we make use of three approaches:

1. $\hat{\beta}$-MSE: As long as the second stage is wellspecified and linear, we can compare the estimated causal parameters $\hat{\beta}$ for the various approaches (where applicable).

2. *FZ/FNZ*: As long as the second stage is wellspecified and linear, we can additionally compare the number of false zero values and false non-zero values to quantify support recovery.

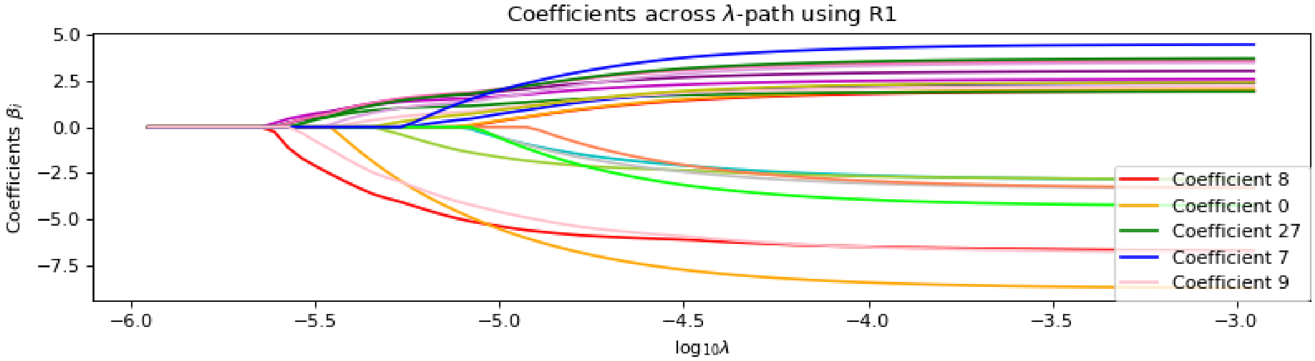

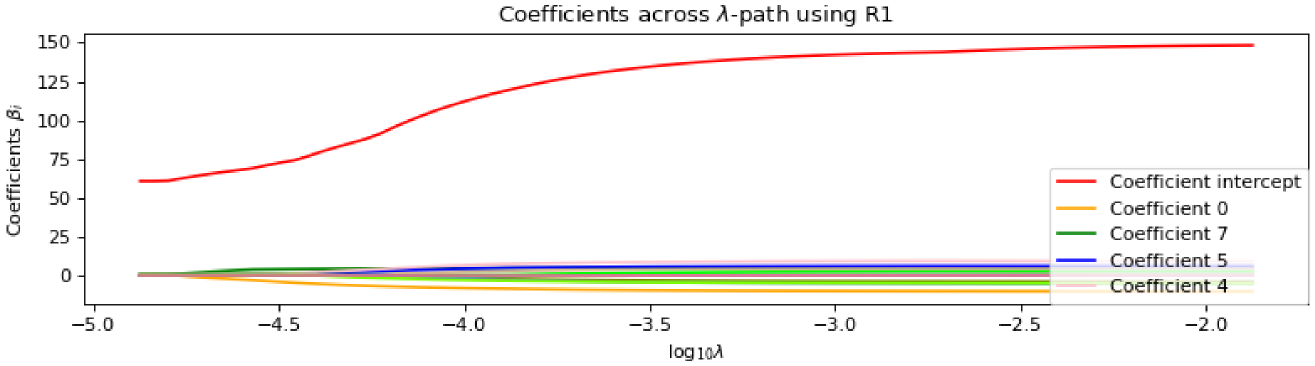

Figure S28. Corresponding λ-path for β coefficients: The plots show the individual coefficients for the different λ values. The upper plot shows the β coefficients for the naive regression, the lower plot presents the coefficients for the two-stage method ILR+LC.

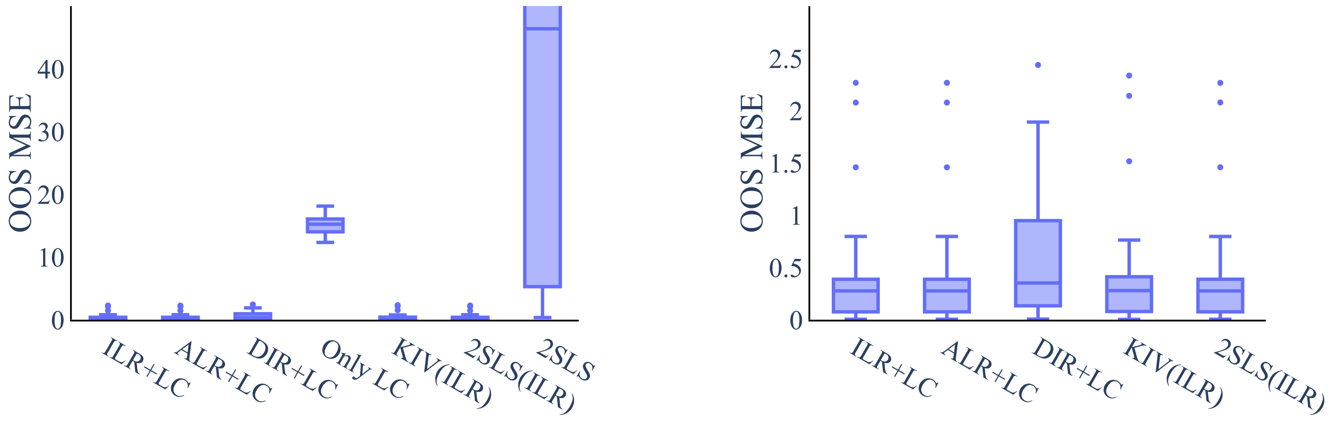

Figure S29. Setting A with $p = 3$, $q = 2$: The boxplots show the OOS MSE of 50 runs. The naive regression Only LC and 2SLS (left) perform way worse compared to the other approaches. When we adjust the y-scale (right), DIR+LC also shows a higher OOS MSE than ILR+LC etc. DIR+LC possibly suffers from the misspecified first stage. Note that ALR+LC,ILR+LC, $2SLS_{ILR}$ are equivalent in the low-dimensional case.

3. *OOS MSE*: In the general case, the causal performance measure is measured by an "out of sample error"(*OOS MSE*) which denotes the mean squared error between the true value of Y under an intervention $do(X = x)$ and the predicted causal effect $\mathbb{E}[Y \mid do(X)]$ of our model, given by $\hat{f}(x)$. For the interventional X, we simulate 250 additional compositional data points according to the underlying model, but using a different seed and thus disconnecting them from the instrument Z and the confounder U. Thus, we receive a true interventional X which still preserves data characteristics.

For each data generating setup, we provide confidence intervals for the methods' results by performing the data generation and the method evaluation 50 times on different random seeds. In each run, we sample $n = 1000$ datapoints in the $p = 3$ scenario and 10,000 datapoints in the $p = 30$ and $p = 250$ scenario. We compute the OOS MSE as well as the $\hat{\beta}$-MSE and FZ/FNZ (if applicable). Some of the figures in this section are extended or more complete versions of the numbers given in the table in the main body (see **????**), where some less relevant results have been omitted for readability.

Setting A

Setting A with $p = 3, q = 2$

This setting is a wellspecified setting for ALR+LC, $2SLS_{ILR}$ and ILR+LC. Moreover, confounding is present (see Figure S7) which additionally gives us reason to expect a much better performance of the two-stage methods than the naive regression $X \to Y$ in terms of OOS MSE. The results in Figure S29, largely verify this expectation. The naive regression has a clear disadvantage due to confounding and picks up on spurious correlations as an effect coming from X. Two-stage methods work well when relying on a strong instrument, helping the methods to factor out the confounding and identifying the true casual effect. Figure S30 shows the causal parameter estimates $\hat{\beta}$ and further corroborates our claims that two-stage methods significantly outperform naive regression. The effects found via naive regression overestimate the direct causal effect strength from X, whereas all two-stage methods recover the true causal parameters β well. Only DIR+LC suffers slightly from the misspecified first stage compared to the other wellspecified two-stage approaches. It is noteworthy that DIR+LC works reasonably well despite our manual two-stage procedure with a "forbidden" non-linear regression in the first stage. Since we are in the low-dimensional setting with no sparsity regularization, the results of ILR+LC, ALR+LC and $2SLS_{ILR}$ are equivalent.

Setting A with $p = 30, q = 10$

Microbiome compositional data is typically high-dimensional and comprises many zero values. Moreover, it is often assumed that only a few microbial compositions (and hence β parameters) influence an outcome of interest Y. Thus, in the following, we aim to be close to such a scenario by assuming a sparse β as ground truth and by simulating X with a few dominating compositions in the data generating process (see Supplementary Material S6).

Note that for higher-dimensional approaches, we omit results for DIR+LC due to computational issues stemming from the maximum likelihood estimation of the α_0 and α parameters in the first stage. 2SLS, which ignores the compositionality of X altogether, is not able to converge at all.

For higher dimensions, the lack of regularization in the ILR methods becomes obvious (Figure S31), both for $2SLS_{ILR}$ and KIV_{ILR}. The methods become more volatile and $2SLS_{ILR}$ is unable to detect any zero values in β (see Figure S32). On the other

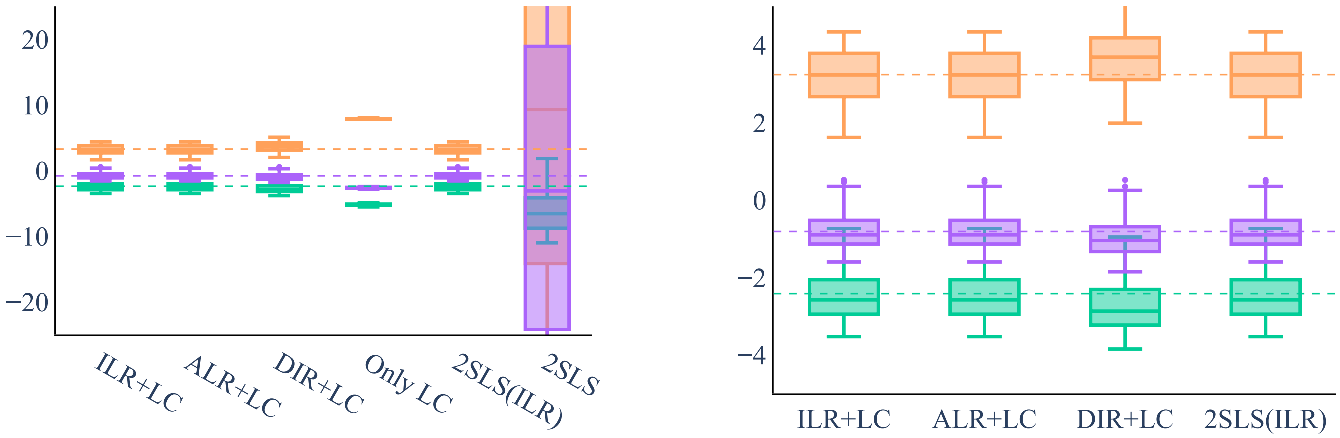

Figure S30. Setting A with $p = 3, q = 2$: The boxplots show the $\hat{\beta}$ values for the 50 runs for each of the 3 β coefficients (dashed lines). The two-stage methods, except 2SLS, are able to recover the causal effect on average. The naive regression method overestimates the effect. Moreover, it does so with a high degree of confidence as there is barely any variation in the $\hat{\beta}$ estimates (left). When we adjust the y-scale (right), DIR+LC shows a notable bias towards the solution of the naive regression (left). This might suggest that DIR+LC indeed suffers from the misspecified first stage and thus is not able to make use of the instrument Z as efficiently.

hand, the naive regression is able to identify zero βs correctly, but suffers from confounding and thus over- or underestimates the true influential βs. Only the regularized two-stage approaches are able to recover the true causal βs, both the influential coefficients as well as the zero values.

Setting A with $p = 250, q = 10$

To further test the approaches, we use another high-dimensional setup with $p = 250$. Again, we make use of the common assumption that only a few microbial compositions (and hence β parameters) influence an outcome of interest Y. We assume a sparse β as ground truth and run the models on X which has a few dominating species.

Note that for higher-dimensional approaches, we omit results for DIR+LC due to computational issues stemming from the maximum likelihood estimation of the α_0 and α parameters in the first stage. 2SLS, which ignores the compositionality of X altogether, is not able to converge at all.

For $p = 250$, the problem of missing regularization in the ILR methods ($2SLS_{ILR}$ and KIV_{ILR}) becomes even more pronounced (Figure S33). For readability we thus omitted $2SLS_{ILR}$ from the β plots. Moreover, the naive regression is not even able to recover the full support, as it only identifies most, but not all, of the zero and non-zero βs correctly (see Figure S32). Only the regularized two-stage approaches are able to recover the true causal βs.

Setting B

In this part we will examine the methods for Setting B **??**. Note that the first stage is misspecified for the two-stage approaches, whereas the second stage is wellspecified for all methods.

Setting B with $p = 3, q = 2$

Even in this low-dimensional scenario, DIR+LC suffers substantially from the misspecified second stage. It is not able to produce sensible estimates. We argue that this might be due to the “forbidden regression” issue. Furthermore, the naive regression is highly influenced by confounding. It even flips the estimated effect of two components, see Figure S36. Nevertheless the remaining two-stage methods, except 2SLS which ignores compositionality, perform reasonably well in recovering the true causal effect (see Figure S35).

Setting B with $p = 30, q = 10$

Microbiome compositional data is typically high-dimensional and comprises many zero values. Moreover, it is often assumed that only a few microbial compositions (and hence β parameters) influence an outcome of interest Y. Thus, in the following, we will emulate such a scenario and assume a sparse β as ground truth and additionally—as ZINegBinom can incorporate sparsity also on X—run the models on relatively sparse X (see Supplementary Material S6).

Note that for higher-dimensional approaches, we omit results for DIR+LC due to computational issues stemming from the maximum likelihood estimation of the α_0 and α parameters in the first stage.

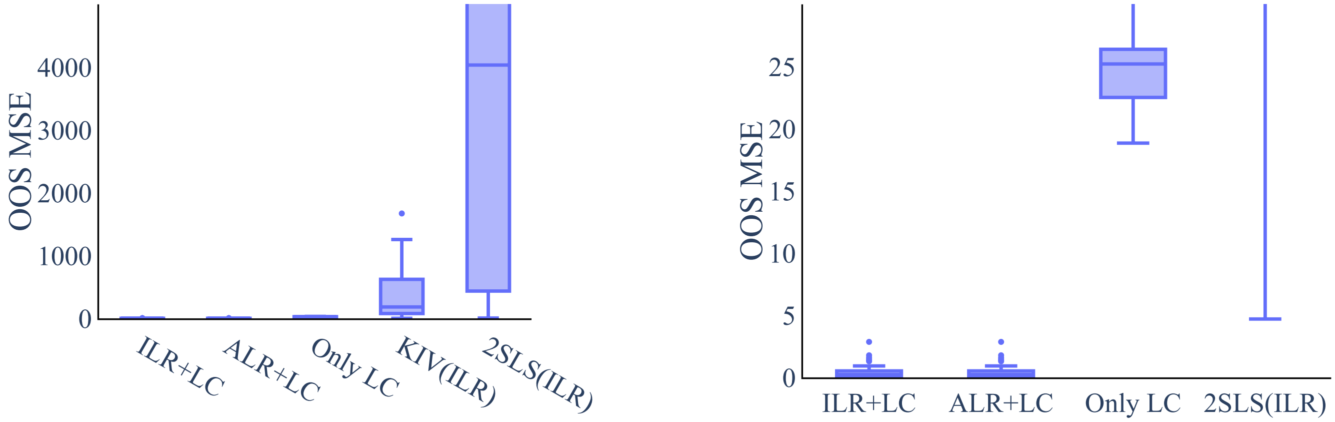

Figure S31. Setting A with $p = 30$**,** $q = 10$**:** The boxplots show the OOS MSE of 50 runs. $2SLS_{ILR}$ and KIV_{ILR} are volatile and lack sensible regularization (left). When we adjust the y-scale, we see that Only LC (right) performs also way worse compared to the regularized two-stage approaches.

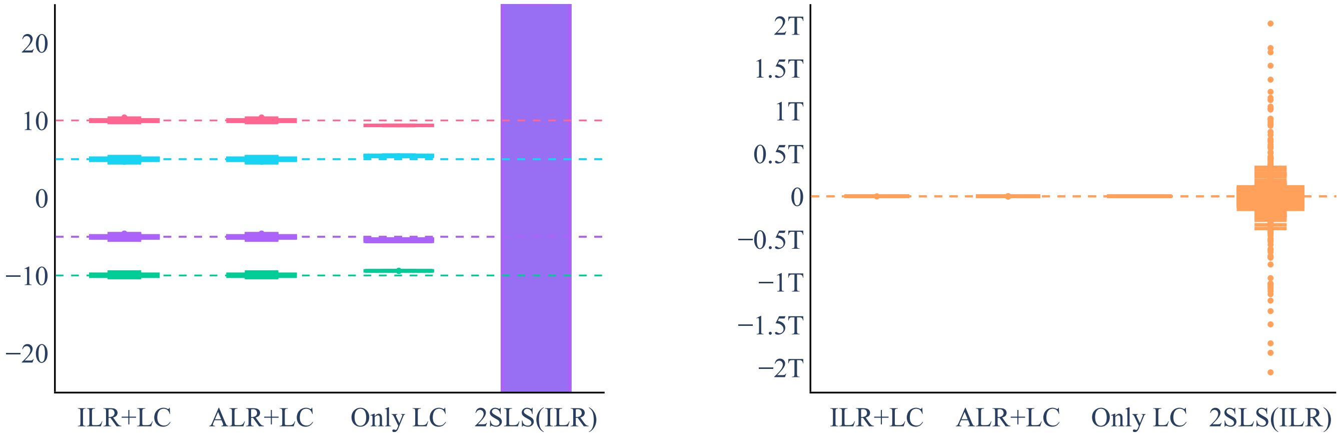

Figure S32. Setting A with $p = 30$**,** $q = 10$**:** The boxplots show the $\hat{\beta}$ values for the 50 runs for each of the 8 non-zero β coefficients (dashed lines, left) and the 22 zero β coefficients (dashed line, right). The two-stage methods are able to recover the causal effect on average, whereas the naive regression methods overestimate the effect (left). Moreover, Only LC does so with a high degree of confidence as there is barely any variation in the $\hat{\beta}$ estimates. $2SLS_{ILR}$ does not produce sensible estimates due to the missing regularization.

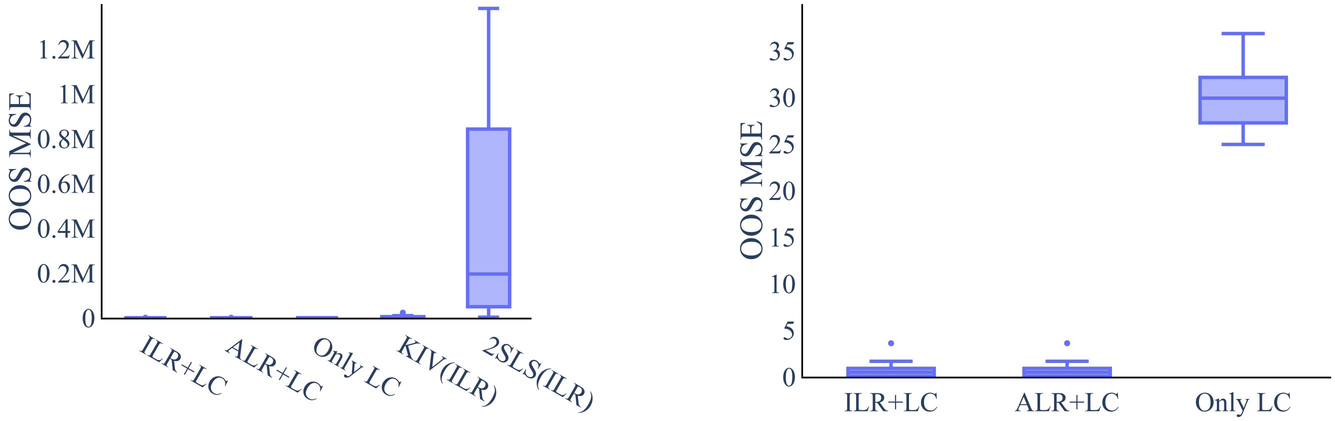

Figure S33. Setting A with $p = 250$**,** $q = 10$**:** The boxplots show the OOS MSE of 50 runs. $2SLS_{ILR}$ and KIV_{ILR} are volatile and lack sensible regularization (left). This problem is more pressing as the dimensionality grows. When we adjust the y-scale, we see that also Only LC (right) performs worse compared to the regularized two-stage approaches.

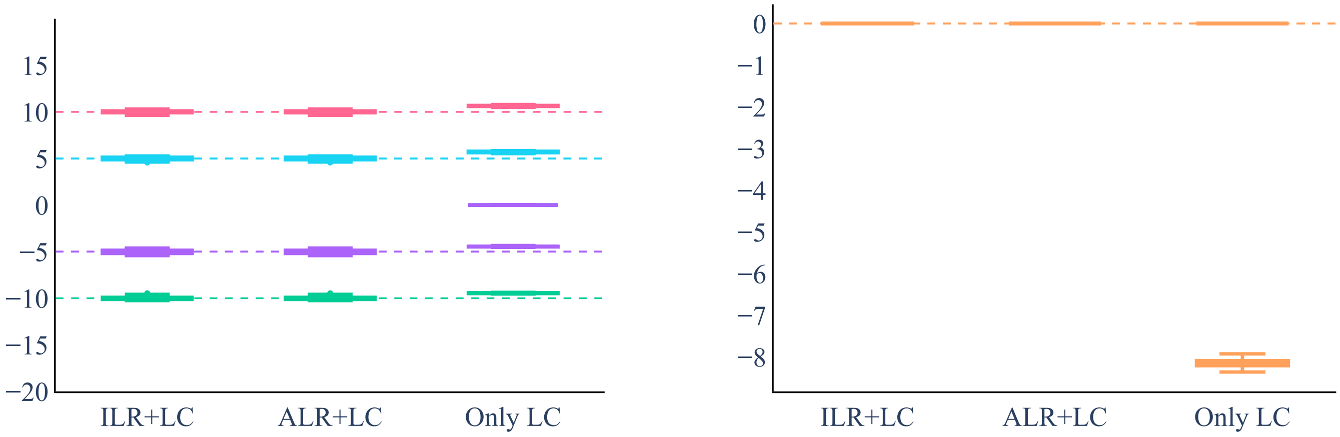

Figure S34. Setting A with $p = 250$, $q = 10$: The boxplots show the $\hat{\beta}$ values for the 50 runs for each of the 8 non-zero β coefficients (dashed lines, left) and the 242 zero β coefficients (dashed line, right). The two-stage methods are able to recover the causal effect on average, whereas the naive regression method is not able to recover the true support. $2SLS_{ILR}$ does not produce sensible estimates due to the missing regularization and is omitted for better readability.

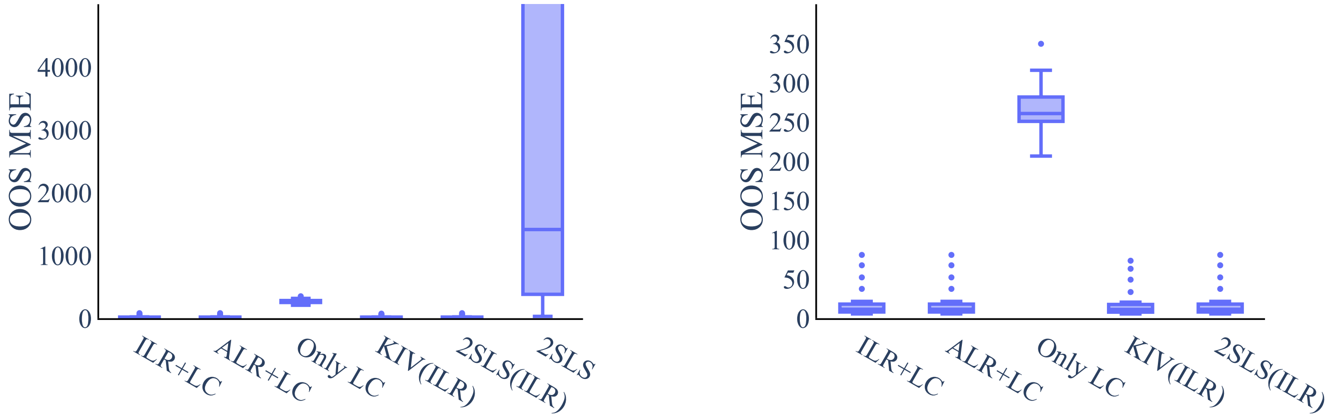

Figure S35. Setting B with $p = 3$, $q = 2$: The boxplots show the OOS MSE of 20 runs. 2SLS and DIR+LC perform way worse as compared to the two-stage approaches (left). When we adjust the y-scale (right), Only LC also cannot compare to the remaining two-stage approaches. ALR+LC, ILR+LC, $2SLS_{ILR}$ are equivalent in the low-dimensional case.

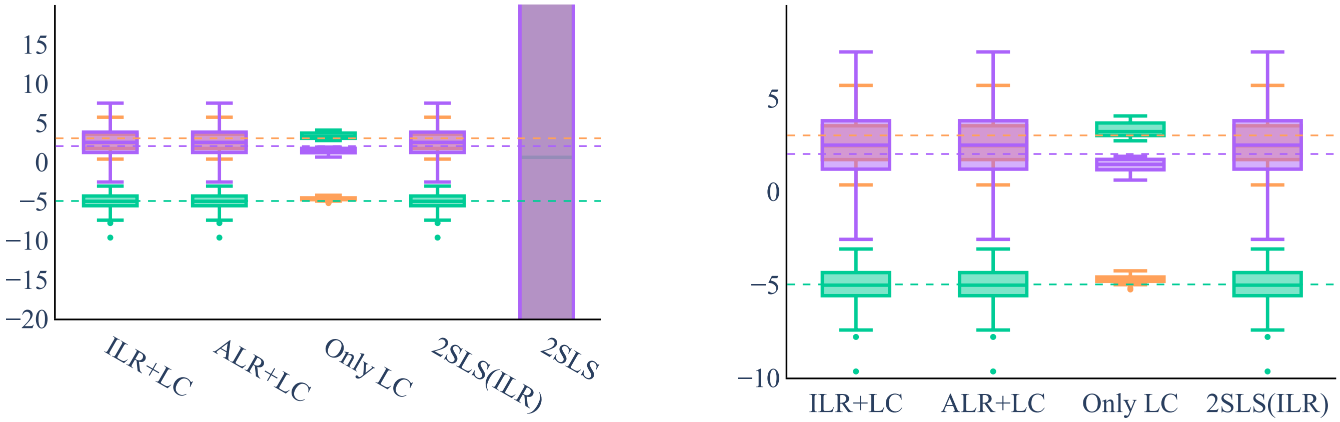

Figure S36. Setting B with $p = 3$, $q = 2$: The boxplots show the $\hat{\beta}$ values for the 20 runs for each of the 3 β coefficients. The two-stage methods (except for DIR+LC and 2SLS) are able to recover the true causal βs on average. However, when we adjust the y-scale (right), the problem of confounding becomes apparent: Only LC flips the sign of two of the non-zero β values.

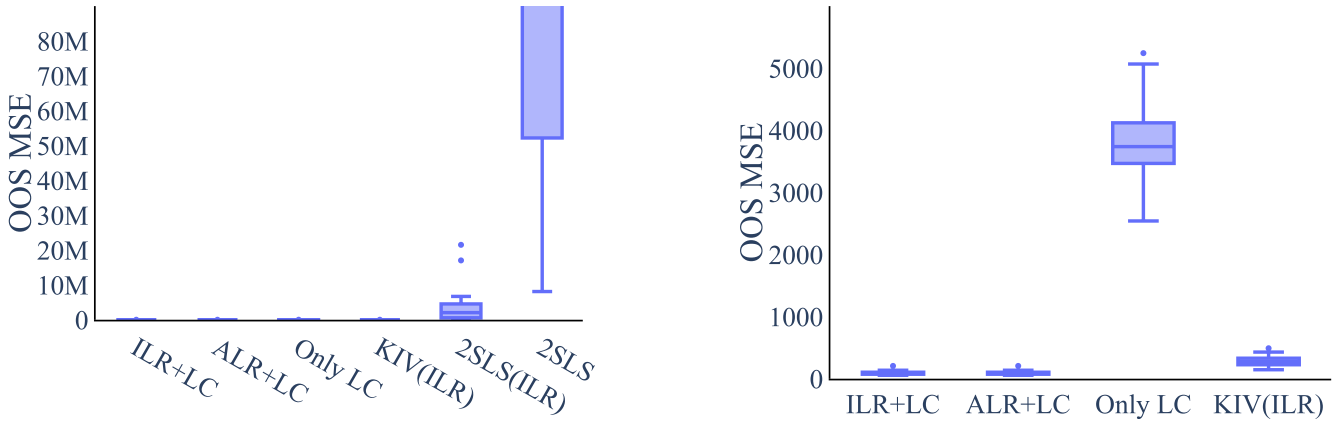

Figure S37. Setting B with $p = 30$**,** $q = 10$**:** The boxplots show the OOS MSE of 20 runs. $2SLS_{ILR}$ is only reasonable in low-dimensions (left). When we adjust the y-scale (right), we see that the remaining two-stage approaches outperform the naive regression.

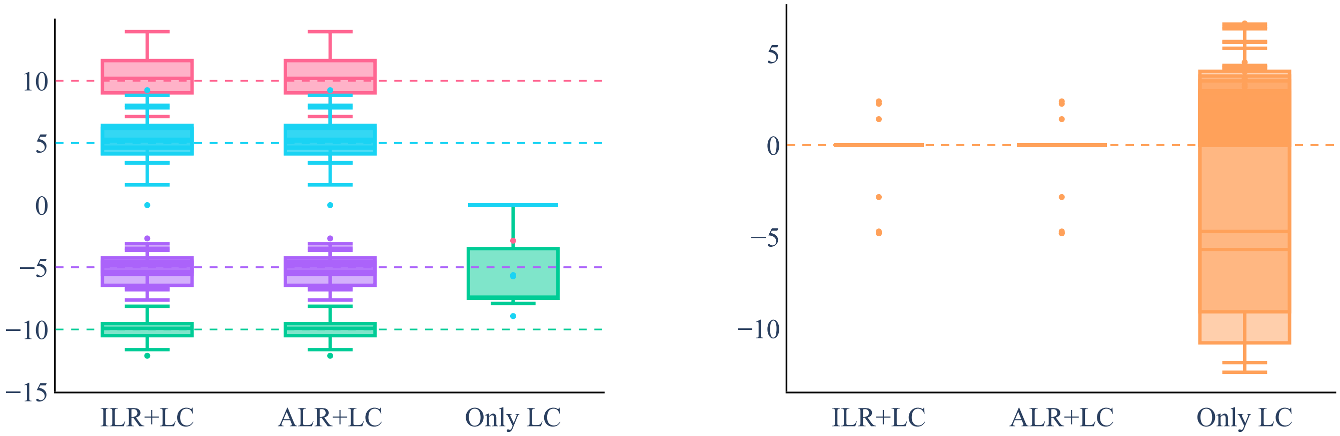

Figure S38. Setting B with $p = 30$**,** $q = 10$**:** The boxplots show the $\hat{\beta}$ values for the 20 runs for each of the 8 non-zero β coefficients (dashed lines, left) and the 22 zero β coefficients (dashed line, right). The naive regression Only LC is not able to recover the β values at all. ILR+LC and ALR+LC are better suited to recover the causal parameters when confounding is present. Despite the misspecified first stage, they are able to recover the causal β values on average.

Moreover, for $p = 30$, $2SLS_{ILR}$ already is unfit to capture the causal effect due to missing regularization. Due to its high OOS MSE value, we omitted $2SLS_{ILR}$ in Figure S38 for better readability. 2SLS, which ignores the compositionality of X altogether, is able to converge, but does not produce reasonable estimates.

For KIV_{ILR}, the difficulty of tuning the method in higher dimensions remains an issue (see Figure S37). The remaining two-stage approaches, however, benefit substantially from the instrumentation of X by Z. They outperform the naive regression both on OOS MSE (see Figure S37), as well as on the recovery of the true β values (see Figure S38). While the naive regression not only fails to recover the true β values, it also produces quite volatile estimates (see Figure S38).

Setting B with $p = 250, q = 10$

Microbiome compositional data is typically high-dimensional and comprises many zero values. Moreover, it is often assumed that only a few microbial compositions (and hence β parameters) influence an outcome of interest Y. Thus, in the following, we will emulate such a scenario and assume a sparse β as ground truth and additionally—as ZINegBinom can incorporate sparsity also on X—run the models on relatively sparse X (see Supplementary Material S6).

Note that for higher-dimensional approaches, we omit results for DIR+LC due to computational issues stemming from the maximum likelihood estimation of the α_0 and α parameters in the first stage.

Both high-dimensional scenarios generally agree in their outcomes; for $p = 250$ the shortcomings of the different approaches only get more enhanced.

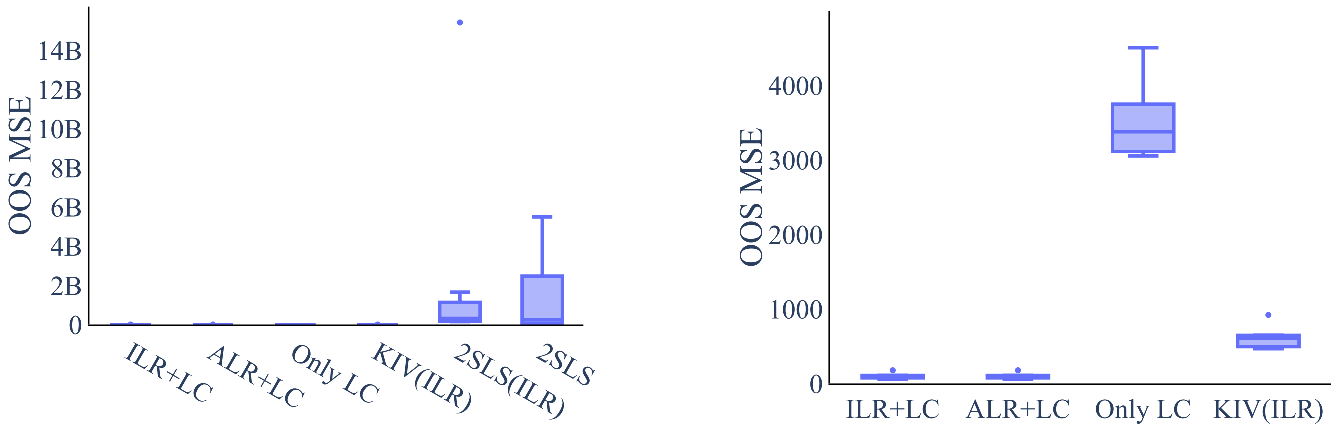

Figure S39. Setting B with $p = 250$, $q = 10$: The boxplots show the OOS MSE of 20 runs. $2SLS_{ILR}$ is only reasonable in low-dimensions and KIV_{ILR} is difficult to tune in higher dimensions (left). When we adjust the y-scale (right), we see that ILR+LC and ALR+LC outperform the naive regression.

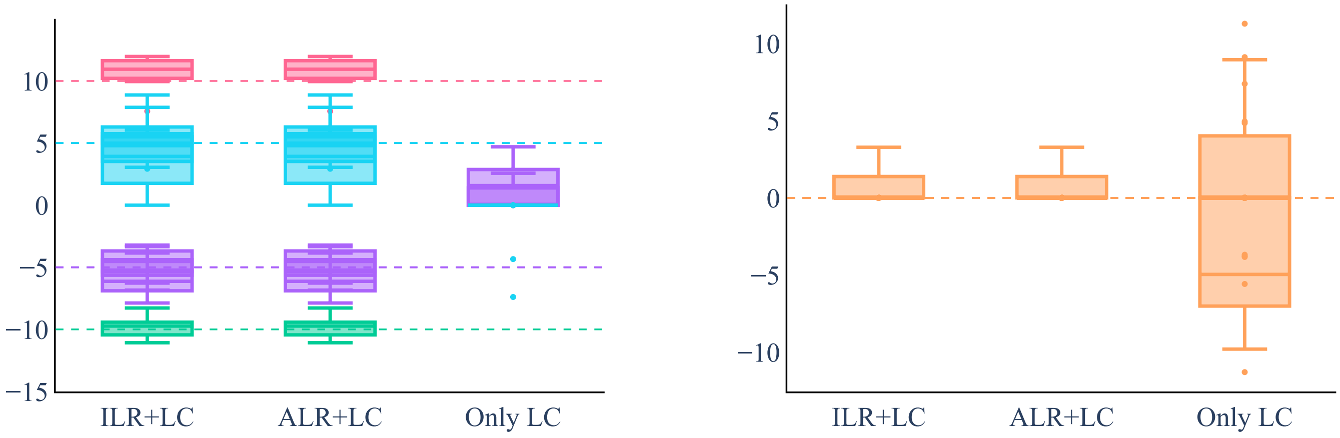

Figure S40. Setting B with $p = 250$, $q = 10$: The boxplots show the $\hat{\beta}$ values for the 20 runs for each of the 8 non-zero β coefficients (dashed lines, left) and the 242 zero β coefficients (dashed line, right). Only LC is not able to recover the β values at all. ILR+LC and ALR+LC are better suited to recover the causal parameters even when confounding is present. Despite the misspecified first stage, they are able to recover the causal β values on average.

While 2SLS, which ignores the compositionality of X altogether, is also able to converge for $p = 250$, it does not produce reasonable estimates. Further, the regularized two-stage methods still perform reasonably well, while $2SLS_{ILR}$ and KIV_{ILR} cannot match that performance (see Figure S39) due to the lack of sensible regularization. The naive approach can capture neither the causal effect nor the causal β values (see Figures S39 and S40).

Further Settings for Robustness Estimation

We will analyze the results form our "robustness" scenarios including a weak instrument setting and a setting with a nonlinear functional relationship in the second stage.

Weak Instrument

Setting A with $p = 3, q = 2$ and weak instruments In a strong/valid instrument setting, two-stage methods have a clear advantage. To test the limitations of our methods, we now analyze an equivalent setting with a comparatively weak instrument. In this setting, confounding is still noticeable (see Figure S21) but the first stage F-statistic is much lower, indicating that we may suffer from weak instrument bias.

The two-stage methods have a higher variation in their estimates, both for OOS MSE and $\hat{\beta}$ (see Figures S41 and S42), whereas the naive regression does not change at all (since only the first stage data generation has changed). Nevertheless, the wellspecified two-stage methods (ALR+LC, ILR+LC, $2SLS_{ILR}$) still recover the causal effects better than the naive regression. Only the DIR+LC regression runs into problems due to two misspecified stages. We thus conclude that the "forbidden

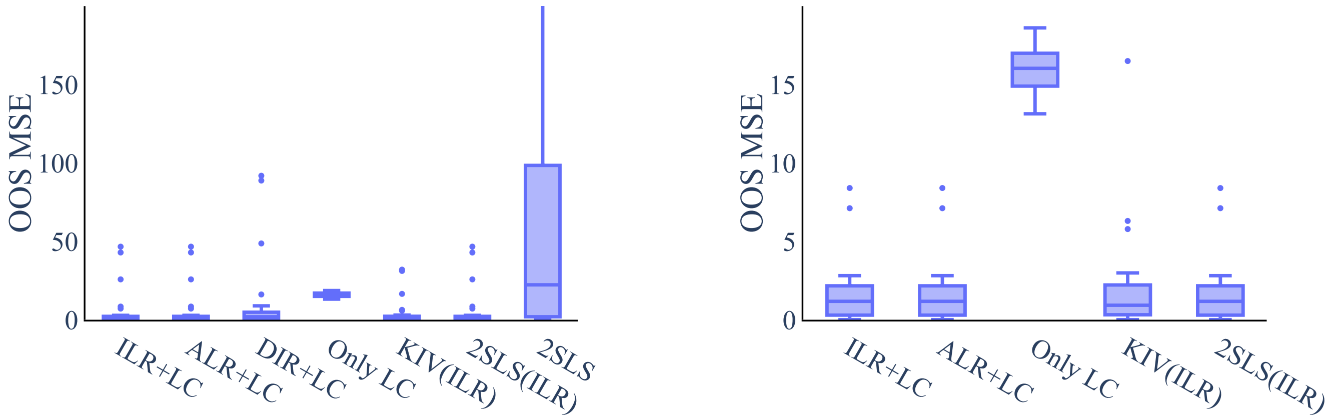

Figure S41. Setting A with $p = 3$, $q = 2$ and weak instruments: The boxplots show the OOS MSE of 50 runs. The DIR+LC performs considerably worse than all the other methods. The problem might stem from the "forbidden regression" issue coming from two misspecified stages. On the right hand side we adjusted the y-scale. The graph shows that the other wellspecified methods still outperform the naive regression in terms of OOS MSE in the weak instrument setting. We observe a higher variance in performance than in the stronger instrument setting.

regression" is not necessarily detrimental to cause-effect estimation when the instrument is strong, but can indeed result in unreliable results for weaker instruments.

Non-linear Second Stage

Setting A with $p = 3, q = 2$ and a non-linear function form of f The two-stage methods perform well if they are in a wellspecified setting. With the DIR+LC method, however, it becomes obvious that misspecification can become problematic. Furthermore, wellspecification is typically impossible to ascertain in practice and most real-world examples are likely not perfectly linear. Thus, we add a polynomial X dependency term in the second stage to evaluate ALR+LC, ILR+LC and $2SLS_{ILR}$ on a partly misspecified setting.

Note that we can only look at the OOS MSE as the β values do not carry any causal interpretation (see Figure S43). The DIR+LC still suffers from two misspecified stages and performs worst. When only the second stage is misspecified, ALR+LC, ILR+LC and $2SLS_{ILR}$ still outperform the naive regressions. However, we are not able to capture the true causal effect because of the misspecification in the second stage. The overall error thus grows in all methods.

Scarce Data Example $p \gg n$

Setting A with $p = 250, q = 10$ and $n = 100$ Also for $n = 100$, the same problem of the lack of regularization persist. $2SLS_{ILR}$ and KIV_{ILR})do not perform well at all (Figure S44). For readability we thus omitted $2SLS_{ILR}$ from the β plots.

As against the large dataset example, the two-stage methods naturally show much larger confidence interval around their estimates, whereas the naive regression does not suffer at the same scale. However the naive regression has troubles to recover the full support (see Figure S45). Thus, even with a much larger uncertainty, the regularized two-stage approaches are able to recover the true causal βs.

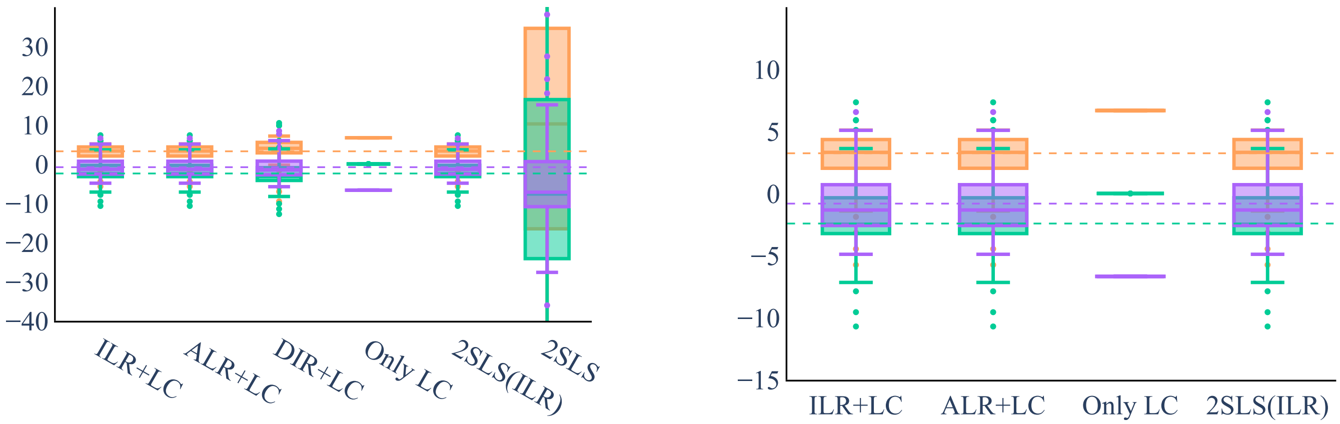

Figure S42. Setting A with $p = 3$, $q = 2$ and weak instruments: The boxplots show the $\hat{\beta}$ values for the 50 runs for each of the 3 β coefficients (dashed lines). In the weaker instrument setting, we are able to recover the true β values for the wellspecified two-stage methods, even though the variance of the $\hat{\beta}$ is higher while the naive regression is still subject to confounding (right, zoomed in plot). Only the DIR+LC is not able to recover the causal effect and seems biased toward the naive regression (left). This might be due to two misspecified stages.

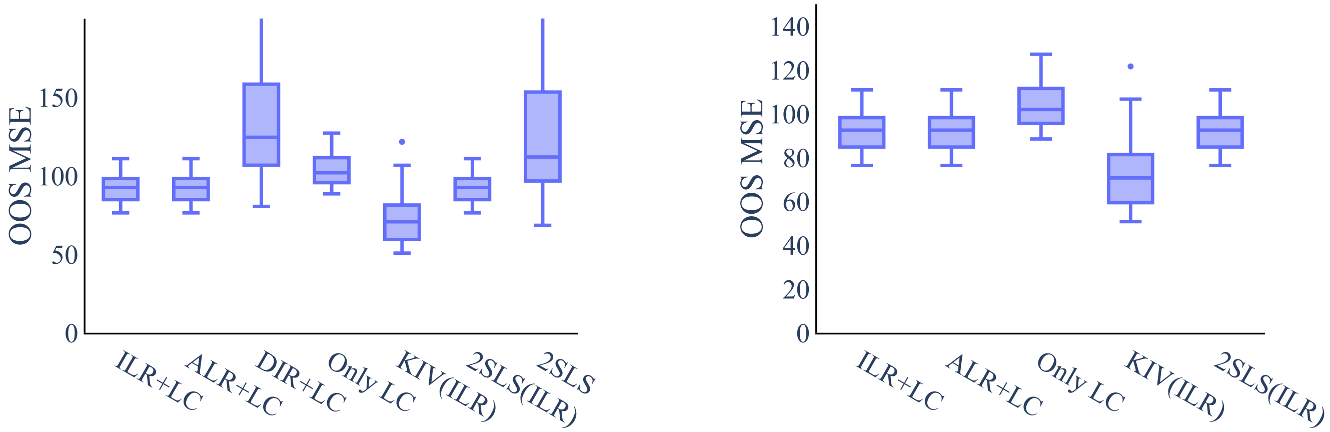

Figure S43. Setting A with $p = 3$, $q = 2$ and a non-linear function form of f: The boxplots show the OOS MSE of 50 runs. DIR+LC and 2SLS perform worst (left). The other two-stage approaches are able to recover the causal effect better than the naive regression. The correctly specified first stage helps in filtering out the confounding effect in the two-stage methods.

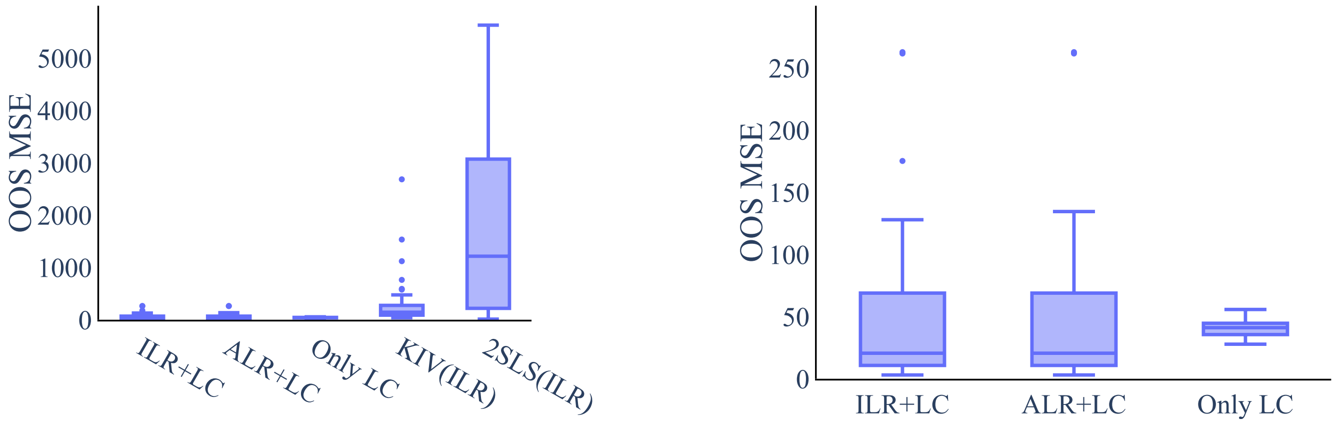

Figure S44. Setting A with $n = 100$, $p = 250$, $q = 10$: The boxplots show the OOS MSE of 50 runs. $2SLS_{ILR}$ and KIV_{ILR} are volatile and lack sensible regularization (left). This problem is more pressing as the dimensionality grows and the number of data samples decreases. When we adjust the y-scale, we see that also Only LC (right) performs worse compared to the regularized two-stage approaches. Note however, that the width of the confidence intervals of the two-stage approaches has increased compared to the larger dataset.

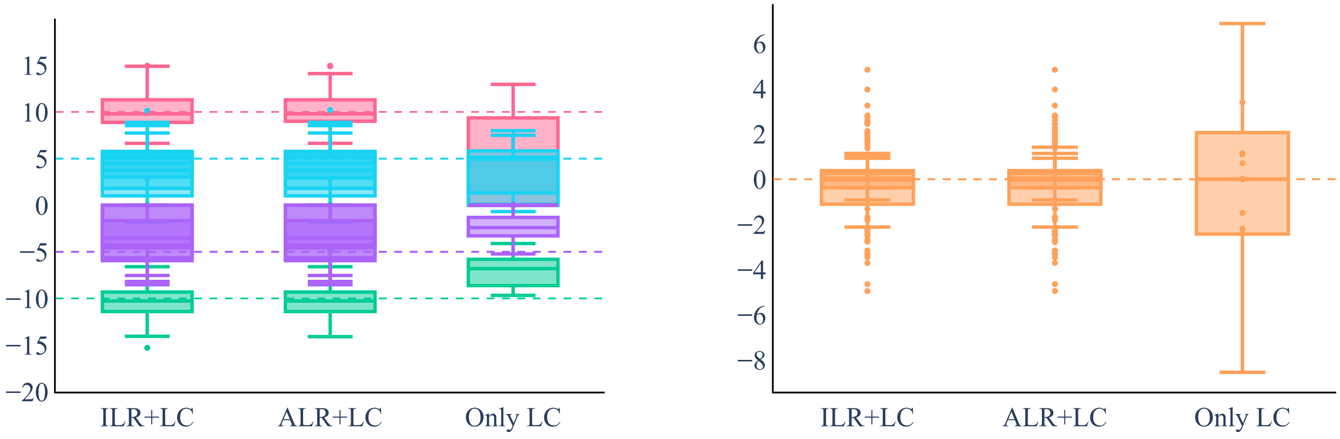

Figure S45. Setting A with $n = 100$, $p = 250$, $q = 10$: The boxplots show the $\hat{\beta}$ values for the 50 runs for each of the 8 non-zero β coefficients (dashed lines, left) and the 242 zero β coefficients (dashed line, right). The two-stage methods are able to recover the causal effect on average, whereas the naive regression method is not able to recover the true support. $2SLS_{ILR}$ does not produce sensible estimates due to the missing regularization and is omitted for better readability. Note however, that the width of the confidence intervals of the two-stage approaches has increased compared to the larger dataset.

References

1. Schulfer, A. *et al.* The impact of early-life sub-therapeutic antibiotic treatment (stat) on excessive weight is robust despite transfer of intestinal microbes. *The ISME J.* **13**, 1, DOI: 10.1038/s41396-019-0349-4 (2019).

2. Rubin, D., Imbens, G. & Angrist, J. Identification of causal effects using instrumental variables: Rejoinder. *J. Am. Stat. Assoc.* **91**, DOI: 10.2307/2291629 (1993).

3. Singh, R., Sahani, M. & Gretton, A. Kernel instrumental variable regression. In *Advances in Neural Information Processing Systems*, 4593–4605 (2019).

4. Kilbertus, N., Kusner, M. J. & Silva, R. A class of algorithms for general instrumental variable models. In *Advances in Neural Information Processing Systems*, vol. 33 (2020).

5. van Rossum, G. & Drake, F. L. *Python 3 Reference Manual* (CreateSpace, 2009).

6. Inc., P. T. Collaborative data science (2015).

7. Harris, C. R. *et al.* Array programming with NumPy. *Nature* (2020).

8. Wes McKinney. Data Structures for Statistical Computing in Python. In Stéfan van der Walt & Jarrod Millman (eds.) *Proceedings of the 9th Python in Science Conference*, 56 – 61, DOI: 10.25080/Majora-92bf1922-00a (2010).

9. Pedregosa, F. *et al.* Scikit-learn: Machine learning in Python. *JMLR* (2011).

10. scikit-bio development team, T. scikit-bio: A bioinformatics library for data scientists, students, and developers (2020).

11. Gautier, L. (2021).

12. Hunter, J. D. Matplotlib: A 2D graphics environment. *Comput. Sci. & Eng.* (2007).

13. Seabold, S. & Perktold, J. statsmodels: Econometric and statistical modeling with python. In *9th Python in Science Conference* (2010).

14. pandas development team, T. pandas-dev/pandas: Pandas, DOI: 10.5281/zenodo.3509134 (2020).

15. Bradbury, J. *et al.* JAX: Composable transformations of Python+NumPy programs (2018).

16. Suh, E. J. (2020).

17. Simpson, L., Combettes, P. & Müller, C. c-lasso - a python package for constrained sparse and robust regression and classification. *J. Open Source Softw.* **6**, 2844, DOI: 10.21105/joss.02844 (2021).

18. R Core Team. *R: A Language and Environment for Statistical Computing*. R Foundation for Statistical Computing, Vienna, Austria (2020).

19. Kurtz, Z. D., Bonneau, R. & Müller, C. L. Disentangling microbial associations from hidden environmental and technical factors via latent graphical models. *bioRxiv* DOI: 10.1101/2019.12.21.885889 (2019).

20. Oksanen, J. *et al.* *vegan: Community Ecology Package* (2020). R package version 2.5-7.

21. Tsagris, M. & Athineou, G. *Compositional: Compositional Data Analysis* (2021). R package version 4.5.

22. Patuzzi, I., Baruzzo, G., Losasso, C., Ricci, A. & Di Camillo, B. metasparsim: a 16s rrna gene sequencing count data simulator. *BMC Bioinforma.* **20**, DOI: 10.1186/s12859-019-2882-6 (2019).

23. Park, J., Yoon, C., Park, C. & Ahn, J. Kernel methods for radial transformed compositional data with many zeros. In *International Conference on Machine Learning*, 17458–17472 (PMLR, 2022).

24. Lin, H. & Peddada, S. D. Multigroup analysis of compositions of microbiomes with covariate adjustments and repeated measures. *Nat. Methods* **21**, 83–91 (2024).

25. Andrews, I., Stock, J. H. & Sun, L. Weak instruments in instrumental variables regression: Theory and practice. *Annu. Rev. Econ.* **11**, 727–753 (2019).

26. Sanderson, E. & Windmeijer, F. A weak instrument f-test in linear iv models with multiple endogenous variables. *J. Econom.* **190**, 212–221, DOI: https://doi.org/10.1016/j.jeconom.2015.06.004 (2016). Endogeneity Problems in Econometrics.
